# Community Characteristics and Potential Risk of Nekton in Waters Adjacent to Ningde Nuclear Power Plant in Fujian, China

**DOI:** 10.3390/biology14050481

**Published:** 2025-04-27

**Authors:** Wen Huang, Biqi Zheng, Dong Wen, Feipeng Wang, Lijing Fan, Zefeng Yu, Wei Liu, Shuang Zhao

**Affiliations:** 1 Ningde Marine Center, Ministry of Natural Resources, Ningde 352100, China; huangwen@ecs.mnr.gov.cn (W.H.); zhengbiqi@ecs.mnr.gov.cn (B.Z.); wendong@ecs.mnr.gov.cn (D.W.); fanlijing@ecs.mnr.gov.cn (L.F.); 2 Key Laboratory of Marine Ecological Monitoring and Restoration Technology, Ministry of Natural Resources, Shanghai 201206, China; 3 Joint Research Center of Marine Ecology of Coastal NPP, Ningde 352100, China; yuzefeng@cgnpc.com.cn; 4 Fujian Key Laboratory on Conservation and Sustainable Utilization of Marine Biodiversity, College of Geography and Oceanography, Minjiang University, Fuzhou 350108, China; fpwang@mju.edu.cn; 5 Fujian Ningde Nuclear Power Co., Ltd., Ningde 355200, China; 6 Ocean College, Fujian Polytechnic Normal University, Fuqing 350300, China; zhaos@fpnu.edu.cn

**Keywords:** nuclear power plants, cooling water systems, nekton, community structure, potentially risky organisms, ecosystems

## Abstract

The impact of bio-invasions and abnormal aggregations of marine life on the safety of cooling water systems in coastal nuclear power plants (NPPs) is significant and cannot be overlooked. In this study, we conducted 12 consecutive monthly surveys from September 2022 to August 2023 in the waters near Ningde NPP in Fujian, China, focusing on nekton species composition, dominant species, abundance, biomass, and diversity indices. We conducted statistical analyses to examine potential correlations between the community structure of these organisms and environmental factors. We recorded 120 species of nekton that belonged to 20 orders, 57 families, and 92 genera, including 72 species of fish, 23 species of shrimp, 19 species of crabs, and 6 species of cephalopods. Pearson and redundancy analyses showed that pH, DIP, and inorganic nitrogen were the main environmental factors driving the observed temporal changes in the nekton community structure in the seawater intake area. We also found that May to October is the peak period for nekton abundance and biomass, and during this time, there is a high risk of nekton blocking the cooling water system of the NPP. These results are of practical significance for NPP managers to prevent and control the clogging of the cooling water system by marine organisms, and the diversity and abundance data provide a theoretical basis for bioecological restoration and management of the area around the Ningde NPP.

## Simple Summary

In recent years, the increasing threat posed by marine organisms to the cooling water systems of coastal power plants has garnered significant attention. In this study, we conducted 12 consecutive monthly surveys in the sea adjacent to the intake of the Ningde nuclear power plant in Fujian, China to investigate the community structure of swimming nekton, such as species composition, dominant species, diversity indices, and their correlation with environmental factors. We documented the main species of swimming nekton, the major influencing environmental factors, and the risks associated with their presence near the cooling source over the course of the year. Our results have practical significance for preventing and controlling the clogging of nuclear power cooling water systems by marine organisms, and our study provides valuable data and a theoretical foundation for ecological restoration and management in the region around the Ningde plant.

## 1. Introduction

With the increasing scarcity of fossil energy resources such as coal and oil, nuclear energy is gaining attention as an efficient and environmentally friendly energy alternative [1,2]. Most coastal power plants are situated in coastal areas and commonly use seawater as the source for the cooling water system [3,4]. The stable operation of the cooling water system, which constitutes a key part of the cooling chain of a nuclear power plant (NPP), is essential for the operational safety of such plants [5,6].

Invasion or abnormal accumulation of marine biota, including nekton, benthos, and plankton, has affected the safety of cooling water systems in coastal NPPs worldwide [5,7,8,9]. For example, in 2003, salps (*Salpa fusiformis*) and krill (*Euphausia pacifica*) obstructed the intake of the cooling water system at the Uljin NPP in South Korea, resulting in a 38% reduction in power generation capacity [10]. The Fitzpatrick NPP and Browns Ferry NPP in the United States and the Koeberg NPP in South Africa were subjected to large influxes of aggregations of fish, which led to a reduction in unit power and an emergency shutdown [11]. Large aggregations of *Acetes* shrimp reduced the safety of the cooling water system at the Lingao NPP and the Yangjiang NPP in China in 2015 and 2016 [12]. In 2014 and 2016, the fish *Stolephorus commersonii* and *Acaudina molpadioidea* damaged the cooling water system at Ningde NPP in China [13]. According to statistics published by the World Association of Nuclear Operators, up to 58% of the nearly 104 cooling water system blockages between 2004 and 2015 were caused by marine biota [14]. Therefore, the prevention and control of marine biota is a top priority for coastal NPPs to reduce the impact of marine biota on water intake.

Currently, the clogging of cooling water systems in coastal NPPs around the world is mainly caused by sea life outbreaks [12,15,16], extreme weather events [17], and accumulation of sea drift [5,18]. To effectively deal with these problems, various monitoring and preventive measures have been proposed, such as monitoring and early warning of catastrophic organism outbreaks [6,16], optimization of the inlet design [5,18], improvement of system resilience [17], and regular cleaning and maintenance of the inlets [5,18]. Hydrodynamic numerical simulation [18,19] offers superior computational simulation; however, the disadvantage is that a large amount of basic data is required. Underwater surveillance systems [15,20,21], capable of delivering remote real-time imagery, face constraints imposed by the complex underwater environment. Acoustic monitoring [22,23], characterized by its extensive coverage, experiences rapid signal degradation and is susceptible to interference. Gene sequencing [24,25], while enabling precise species identification, is hindered by its time-consuming and high costs. These technologies are mainly targeted at benthic organisms [26], jellyfish [27,28,29,30,31], phytoplankton [32,33], *Acetes* [34], fouling organisms [35,36,37], suspended sediments [38], sea ice [39,40], and other clogging agents.

The impact of marine biota on cooling water systems has gradually attracted the attention of NPPs and safety authorities. Relevant research on benthic organisms [26,41], jellyfish [42], zooplankton [43], and phytoplankton [44] has been conducted, but there is insufficient understanding of the species composition, abundance, spatial and temporal variations, and relationships with environmental factors of the nekton in the sea area adjacent to the Ningde NPP. Therefore, we conducted the first monthly monitoring of the nekton in the sea adjacent to this NPP for one year and analyzed the species composition, dominant species, diversity indices, abundance, biomass, and their correlations with environmental factors. We used the data to identify organisms that potentially pose a safety risk to the cooling water system of the plant and to propose suggestions for the prevention and control of blockages by these organisms. Additionally, the diversity and abundance data provide a theoretical basis for bioecological restoration and management of the area around the Ningde NPP.

## 2. Materials and Methods

### 2.1. Sample Collection, Identification, and Classification

**Figure 1:**
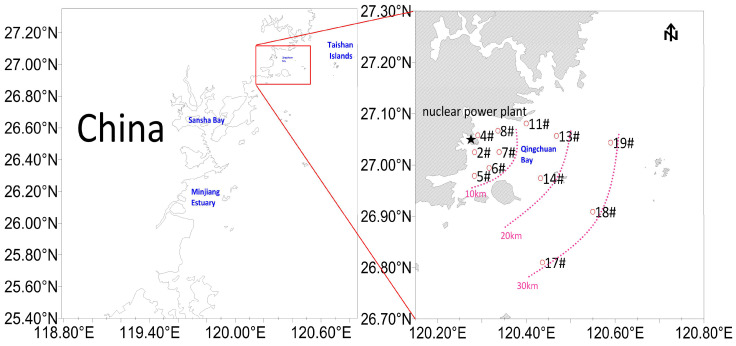
Survey stations in the seawater intake area of the Ningde NPP. Note: The pink dotted line indicates the distance from the cooling water system of Ningde NPP. The ★ symbol indicates the location of the Ningde NPP. Numbers with # denote different sampling stations (2#, 4#, 5#, 6#, 7#, 8#, 11#, 13#, 14#, 17#, 18#, 19#).

Twelve stations (120.14° E–120.80° E, 26.70° N–27.30° N) (2#, 4#, 5#, 6#, 7#, 8#, 11#, 13#, 14#, 17#, 18#, 19#) were sampled within 30 km of the NPP seawater intake area during spring and autumn surveys of nekton and water quality (Figure 1), and two of these stations (6# and 7#, 10 km from the NPP) were sampled monthly from September 2022 to August 2023. Nekton sampling was conducted aboard the “Min Fuding Yu 02786 vessel” using a single capsule bottom trawl with a 20 mm mesh size. The average trawl operation time per station was 0.5 h, and the trawl speed was maintained at 2–3 kn. Samples were refrigerated and brought back to the laboratory for immediate classification, identification, and measurement.

Fish classification and identification were performed following the Marine Fishes of China [45], and crab and shrimp classification and identification were conducted following the East China Sea Economic Shrimp and Crab [46] and Atlas of Chinese Marine Biota (Vol. VI) [47]. The family Squillidae was classified as shrimp for statistical analysis. Cephalopods identification was conducted following Donghai Economic Cephalopods [48].

### 2.2. Measurements of Environmental Variables

At each station, water temperature (temp), salinity (sal), and pH were measured on-site using a multi-parameter water quality analyzer (CTD, 1481, Sea and Sun Technology, Trappenkamp, Germany). Water samples were brought back to the laboratory to measure chemical oxygen demand (COD) and contents of dissolved oxygen (DO), dissolved inorganic nitrogen (DIN), dissolved inorganic phosphorus (DIP), and suspended solids (SS). The water quality surveys were conducted in accordance with the “Ocean Monitoring Specification” (GB17378.3-2007) [49,50].

### 2.3. Data Processing and Analysis

The index of relative importance (IRI) [51] was used to calculate the species dominance of the nekton community. The diversity of nekton was analyzed using Shannon–Wiener’s diversity index (*H*), Pielou’s evenness index (*J*), and Margalef’s species richness index (*D*) [52], and the trawl sweeping area was used to estimate the density of the nekton [53]. The nekton survey was conducted based on the standard Technical Procedures for Evaluation of the Impact of Construction Projects on Marine Biota Resources (SCT9110-2007) [54,55]. The formulas used are as follows:

IRI = (N% + W%) × F% × 10^4^ (1)

*D* = (S − 1)/lnN (2)

(3) $$H=-\sum_{i=1}^{S}\mathrm{Pi}\times \ln\mathrm{Pi}$$

*J* = *H*/lnS (4)

ρi = (Ci/Ai) × q(5)

For IRI, N% and W% are the percentage of number and percentage of weight of the species in the catch, respectively, and F% is the percentage frequency of occurrence of the species. Species with IRI > 500 are considered to be dominant [56]. For the other indexes, S is the number of species, N is the total number of nekton individuals in the sample, Pi is the ratio of the number of individuals of species i to the total number of individuals, ρi is the density of the nekton at station i (kg/km^2^ or ind/km^2^), C_i_ is the hourly trawl catch at station i (kg/h or ind/h), Ai is the hourly trawl swept sea area at station i (km^2^/h), and q is the trawl catch rate, which was set to 0.5 in this study.

Parametric assumptions were systematically validated through Kolmogorov–Smirnov normality testing for environmental and nekton variables, complemented by Levene’s homoscedasticity evaluation, with subsequent model residuals examined (Tables S1–S9, Figures S1–S42). When the assumptions of homoscedasticity were not met, parameters of abundance, biomass, and environmental variables were logarithmically [log_10_(x + 1)] transformed. The season was treated as a fixed factor, with monthly diversity index (*H*, *J*, and *D*) values nested within a season (using 6# and 7# stations). A nested Analysis of Variance (ANOVA) followed by Fisher’s least significant difference (LSD) post hoc test was conducted to assess the statistical differences in the nekton species diversity index (Tables S10–S15, Figures S43–S45). The nested ANOVA followed the theory proposed by Underwood [57], with reference to the method used by Jonathan [58]. The significance levels were adjusted using the Bonferroni correction to account for multiple comparisons. All of the statistical analyses were performed using SPSS 27 (https://www.ibm.com/spss (accessed on 12 December 2024)). The sampling periods were defined as follows: Autumn (September, October, and November), Winter (December, January, and February), Spring (March, April, and May), and Summer (June, July, and August).

Pearson correlation analysis and redundancy analysis (RDA) were employed to evaluate the relationship between nekton and environmental factors. The Pearson correlation analysis results were calibrated by the Bonferroni test to avoid accidentally significant results. Before conducting RDA, we performed a DCA (Detrended Correspondence Analysis) analysis, and the results showed that the longest axis was 3.32, which is less than 4.00. This suggests that the relationship between environmental variables and the nekton community is relatively linear, so we chose RDA for further analysis. A Monte Carlo permutation was employed to test the significance of environmental variables in explaining the nekton abundances under an unrestricted model of 999 permutations. All of these analyses were conducted using the Vegan package in R (version 4.10) [59]. Survey station diagrams were produced using Surfer 16 (https://www.goldensoftware.com (accessed on 12 December 2024)).

## 3. Results

### 3.1. Nekton Species Collected During the Surveys

**Figure 2:**
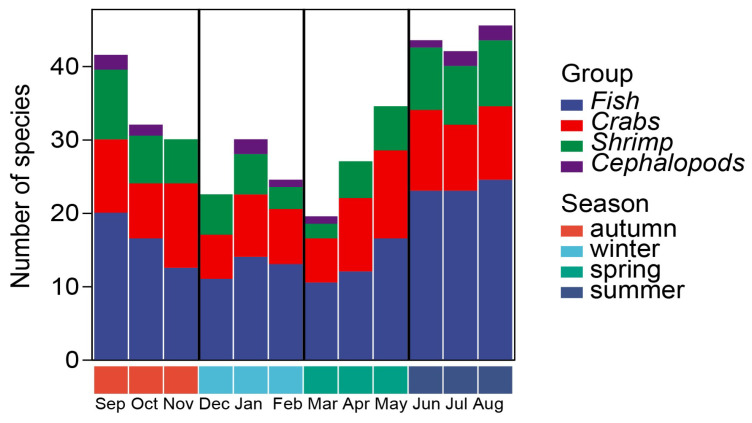
Annual variation in species numbers of various nekton groups.

During the trawl survey from September 2022 to August 2023, 120 species of nekton belonging to 20 orders, 57 families, and 92 genera, were recorded in the seawater intake area of the NPP. The distribution of the nekton groups (Figure 2) showed that fish species were the most abundant, with 72 species that accounted for 60.00% of the total number of species. They were distributed in 12 orders, 39 families, and 60 genera. Shrimp species were the second most abundant, with 23 species that accounted for 19.17% of the total number of species. They belonged to 2 orders, 8 families, and 20 genera. Nineteen species of crabs were collected, accounting for 15.83% of the total number of species. They belonged to two orders, six families, and eight genera. Cephalopods were the least numerous. The six species accounted for 5.00% of the total number of species and belonged to four orders, four families, and four genera.

### 3.2. Seasonal Changes in Dominant Species of Nekton

Based on an IRI score > 500 (Table 1), there were three dominant species common to the four seasons: mantis shrimp (*Oratosquilla oratoria*), gazami crab (*Portunus trituberculatus*), and branded goby (*Chaeturichthys stigmatias*). These three species and the lizardfish (*Harpodou nehereus*) were dominant in spring, summer, and autumn.

In spring, the 14 dominant species included croaker (*Collichthys lucidus*), burrowing goby (*Trypauchen vagina*), *O. oratoria*, *C. stigmatias*, and others, and their biomass accounted for 76.53% of the total biomass. In summer, the 13 dominant species were *O. oratoria*, *P. trituberculatus*, white croaker (*Argyrosomus argentatus*), and *H. nehereus* and others, and their biomass accounted for 72.22% of the total biomass. Nineteen species were dominant in autumn, including *O. oratoria*, *H. nehereus*, white shad (*I. elongata*), and anchovies (*T. mystax*) with a mid-maxilliped. Their biomass accounted for 84.54% of the total biomass. In winter, the 13 dominant species included *O. oratoria*, *H. nehereus*, *I. elongata*, and *T. mystax*, and the biomass of these species accounted for 81.60% of the total biomass.

### 3.3. Seasonal Changes in Nekton Diversity Indexes

**Figure 3:**
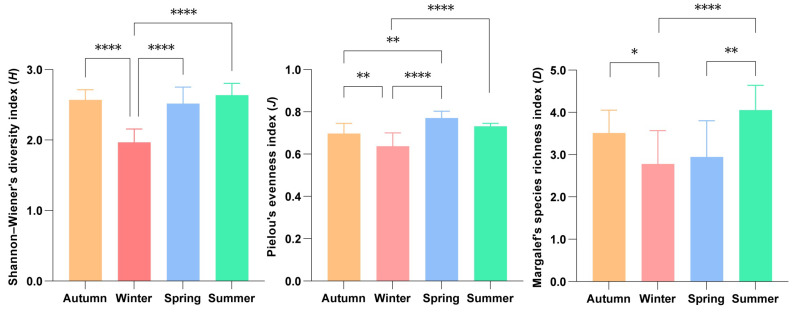
Seasonal variation of nekton diversity indexes. Note: * indicates significance *p* < 0.05, ** indicates significance *p* < 0.01, **** indicates significance *p* < 0.001.

Figure 3 shows the seasonal variation of nekton diversity. Nested ANOVAs showed *H* in autumn was significantly higher than that in winter (*p* < 0.001), but did not differ significantly from that in spring and summer (*p* > 0.05) (Tables S10 and S11). Winter had the lowest diversity value (1.80), which was significantly lower than that of spring (*p* < 0.001) and summer (*p* < 0.001). Nested ANOVAs showed *J* in autumn was significantly higher than that in winter (*p* < 0.01), but significantly lower than that in spring (*p* < 0.01) (Tables S12 and S13). Winter had the lowest evenness value (0.57) of the year, which was significantly lower than that in autumn (*p* < 0.01), spring (*p* < 0.001) and summer (*p* < 0.001). There was no significant difference in evenness between spring and summer (*p* > 0.05). Nested ANOVAs showed *D* in summer was significantly higher than that in winter (*p* < 0.001) and spring (*p* < 0.01) (Tables S14 and S15). Winter had the lowest richness value (2.23), which was significantly lower than that of autumn (*p* < 0.05). The richness in autumn did not differ significantly from that in spring (*p* > 0.05) and summer (*p* > 0.05). These results indicate that winter exhibited lower *H* and *J*, while summer showed higher *D* compared to other seasons.

### 3.4. Seasonal Changes in the Volume of Nekton

**Figure 4:**
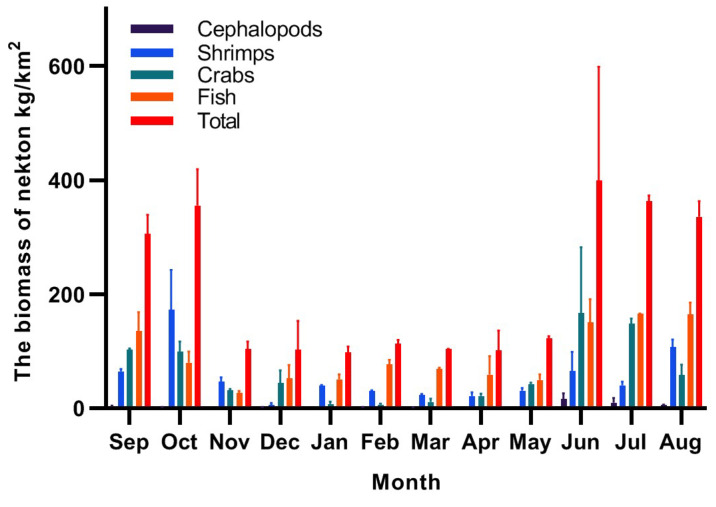
Seasonal changes in the biomass of nekton.

**Figure 5:**
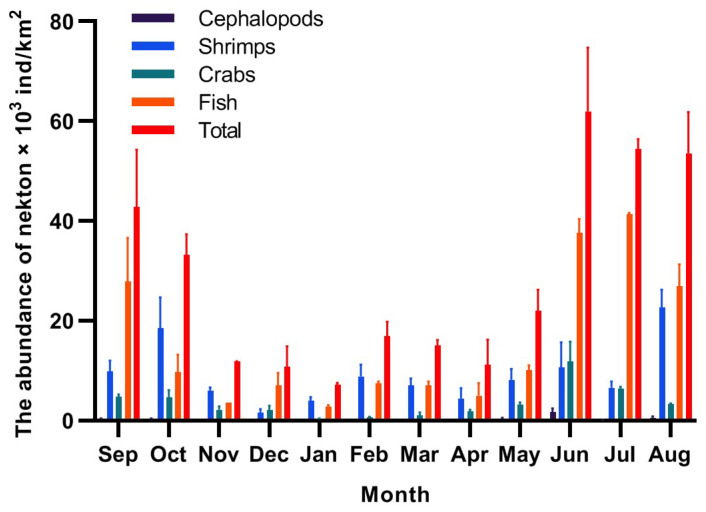
Seasonal changes in the abundance of nekton.

Figure 4 and Figure 5 show the monthly changes in the biomass and abundance of nekton, respectively, in the seawater intake area of the NPP. The monthly mean biomass was 212.06 kg/km^2^, and the monthly mean abundance was 28.54 × 10^3^ ind/km^2^. Both values were highest in June and lowest in January. Fish constituted the main component of nekton biomass, and the monthly mean biomass accounted for 43.55% of the total monthly mean biomass. Shrimp and crabs accounted for 25.42% and 28.96%, respectively, and cephalopods accounted for the lowest proportion of total mean biomass. The main constituent groups of nekton abundance were fish, shrimp, crabs, and cephalopods, with proportions of 52.39%, 31.60%, 14.79%, and 0.93%, respectively.

The most dominant species in autumn and winter was *O. oratoria*. Its average biomass and abundance in autumn were 63.67 kg/km^2^ and 5.83 × 10^3^ ind/km^2^, respectively. The maximum value appeared in October, during which the biomass and abundance were 140.53 kg/km^2^ and 14.28 × 10^3^ ind/km^2^, respectively. In winter the average biomass of *O. oratoria* was 19.24 kg/km^2^, and the mean abundance was 1.42 × 10^3^ ind/km^2^.

*T. uagina* was the most dominant species in spring, with a mean biomass of 16.84 kg/km^2^ and a mean abundance of 1.60 × 10^3^ ind/km^2^. The maximum values occurred in April, when the mean biomass and abundance were 40.64 kg/km^2^ and 3.69 × 10^3^ ind/km^2^, respectively. The maximum dominance of *A. argentatus* reached 5001 (IRI index) in summer (June–August), with a mean biomass of 39.98 kg/km^2^ and a mean abundance of 13.24 × 10^3^ ind./km^2^ in summer, and the maximum values occurred in June, with a biomass of 68.80 kg/km^2^ and an abundance of 22.46 × 10^3^ ind./km^2^, respectively.

### 3.5. Spatial Distribution of Nekton and Its Correlation with Environmental Factors

**Figure 6:**
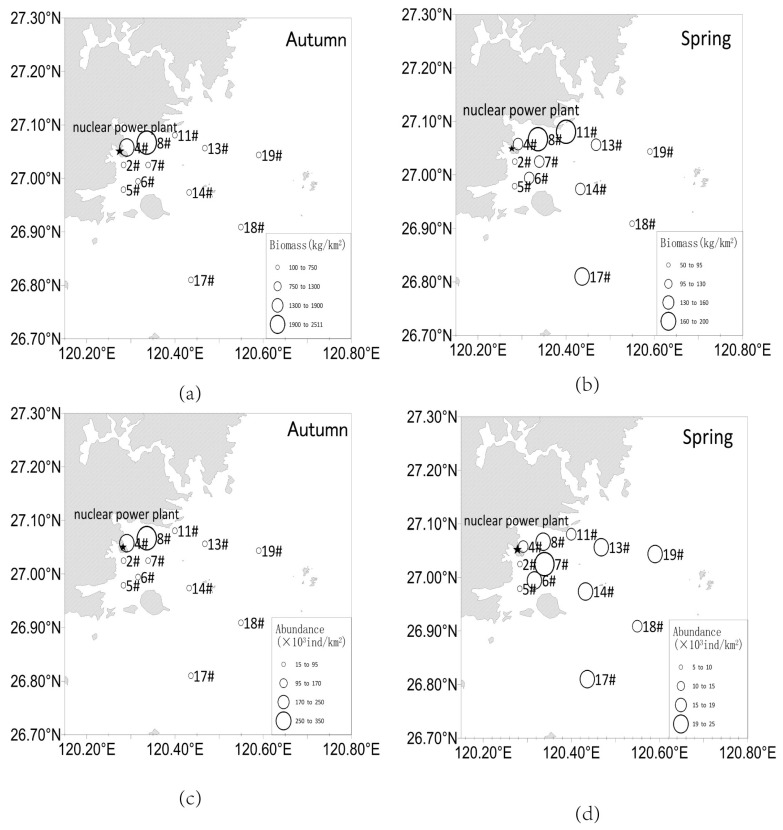
Distribution of nekton biomass and abundance in spring and autumn: (**a**) Nekton biomass in autumn; (**b**) Nekton biomass in spring; (**c**) Nekton abundance in autumn; (**d**) Nekton abundance in spring. The ★ symbol indicates the location of the Ningde NPP. Numbers with # denote different sampling stations (2#, 4#, 5#, 6#, 7#, 8#, 11#, 13#, 14#, 17#, 18#, 19#).

We plotted the distribution of nekton biomass and abundance at each station in the NPP seawater intake area (Figure 6). The biomass and abundance of nekton in autumn ranged from 154.15 kg/km^2^ to 2510.56 kg/km^2^ and 18.54 × 10^3^ ind/km^2^ to 32.40 × 10^3^ ind/km^2^, respectively, with the maximum values of both in autumn occurring at station 8#. In spring, nekton biomass and abundance ranged from 63.49 kg/km^2^ to 197.17 kg/km^2^ and 5.92 × 10^3^ ind/km^2^ to 23.96 × 10^3^ ind/km^2^, respectively, with the maximum biomass value occurring at station 8# and the maximum abundance occurring at station #7. Overall, the biomass and abundance were higher in autumn than in spring. Moreover, the values of both were higher at stations in closer proximity to the NPP, which further indicates that this sea area is rich in nekton, which poses a potential safety risk to the plant’s cold water source.

**Figure 7:**
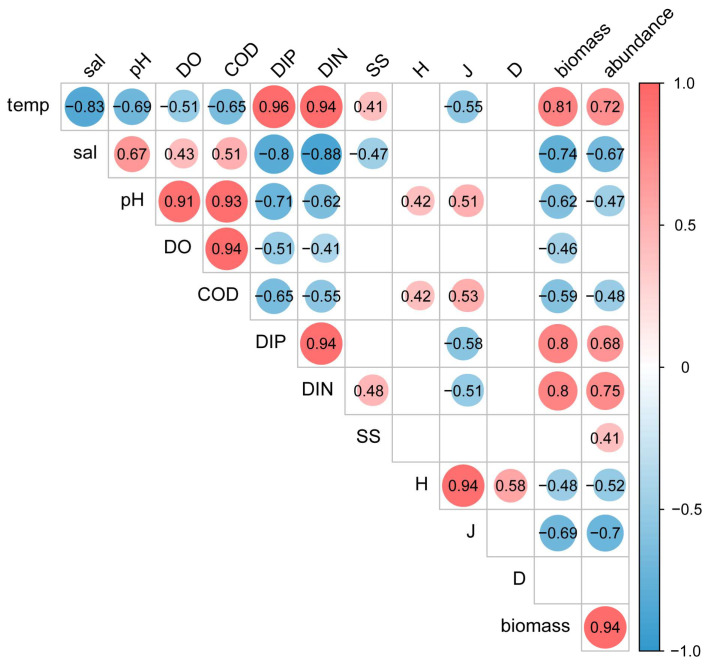
Pearson analysis of nekton biomass, abundance, and environmental factors in spring and autumn.

Pearson correlation analyses (Figure 7) between the biomass, abundance of the nekton and The parameters (*H*, *J*, *D*, temperature, salinity, pH, DO, COD, DIP, DIN, and SS) revealed highly significant positive correlations between nekton biomass and abundance versus water temperature, DIP, and DIN (r > 0.68, *p* < 0.01) and a highly significant negative correlation (r < 0.47, *p* < 0.01) between abundance and biomass versus salinity, pH, and COD.

**Figure 8:**
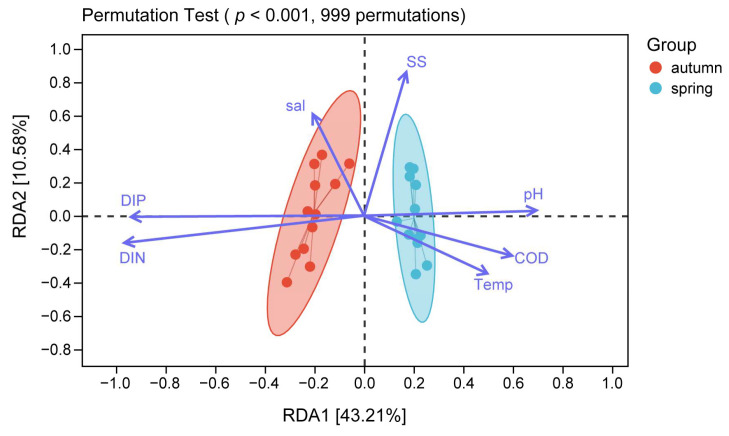
RDA analysis of community structure and environmental factors for nekton in spring and autumn.

The amount of nekton and habitat distribution are often affected by a variety of factors such as temperature, salinity, and pH [60,61,62,63], so we used multivariate RDA to analyze the relationship between nekton and environmental factors. The Monte Carlo permutation test (*p* < 0.001, 999 permutations) was used to select the high-impact environmental factors for plotting (Figure 8). The first sorting axis explained 43.21% of the variance, while the second axis accounted for an additional 10.58%. Together, the first two axes cumulatively explained 53.79% of the variance in the relationship between the nekton community and the environmental factors. Moreover, the results suggested that pH, DIP, and DIN are the main environmental factors influencing the structure of the nekton community.

## 4. Discussion

### 4.1. Composition of the Nekton Community

The formation of biological community structure is based on species composition, and the fishery resources in the sea area around the Ningde NPP are reflected by the species composition of nekton [64]. We collected 120 species of nekton in this survey, which consisted of 72 species of fish, 23 species of shrimp, 19 species of crab, and 6 species of cephalopods. During the consecutive 12-month survey, the nekton species were dominated by fish, indicating that fish are the main taxa in this sea area. Compared with neighboring areas, the number of nekton species in the Ningde NPP intake waters was higher than that in Oujiang River Estuary in Zhejiang [53] (78 species of nekton, 36 species of fish, 28 species of crustaceans, and 3 species of cephalopods) and Qixing Island waters [65] but lower than that in Minjiangkou waters (125 species of fish in 13 orders) [66] and Sansha Bay waters (94 species of fish) [67]. A comparison of different groups revealed that all of these sea areas, including that around the Ningde NPP, were dominated by fish and then crustaceans, with cephalopods being the least abundant.

A comparison of historical survey data (Table 2) revealed that the number of nekton species in the study area fluctuated from 2012 to 2023, with a decrease and then an increase. There are three possible reasons: First, the survey method in this study was once a month in an annual period, while the survey method in other areas [52,53,65,67] was mostly in spring and autumn, ignoring the changes of other seasons. Second, there are slight differences in the use methods of the research investigation; Shen [67] used a 1.7 cm mesh trawl operation for 0.33 h, while this study and other studies [52] used a 2.0 cm mesh trawl operation for 0.5 h, and Ke et al. [68] used 2.5 cm~1.3 cm mesh trawl operation for 0.5–1.0 h, and Song et al. [53] used 2.0 cm mesh trawl operation for 0.5 h–1.0 h. Third, the research stations and investigation areas were different, resulting in differences in the number of nekton species.

The number of nekton species detected in our study was lower than that in the offshore waters of the Taishan Islands [67] but higher than that in the waters of the Qixing Islands [65] and the waters of Qingchuan Bay [52], suggesting that the number of nekton species showed a significant temporal fluctuation. This change may be caused by a combination of natural changes in the marine environment, disturbances from human activities, and shifts in material cycling and energy flow within the ecosystem.

The nekton consisted only of warm-water and warm-temperature species, as no cold-temperature or cold-water species were found, which is in line with the environmental characteristics of subtropical seas. Temporally, the number of nekton species peaked in August (46 species) and was lowest in March (20 species). In terms of different groups, the highest number of fish species occurred in August (25 species) and the lowest in March (10.5 species), crabs were most and least abundant in November (12 species) and March (6 species), respectively, shrimp species peaked in September (10 species) and were lowest in March (2 species), and the number of cephalopods did not change much from month to month. Overall, the number of nekton species showed a pattern of summer > autumn > spring > winter and the composition pattern was fish > shrimp > crabs > cephalopods.

Temporal changes in the environment likely explain the temporal pattern of nekton diversity and abundance. In summer and autumn, the southeast monsoon gradually strengthened, the coastal currents of Zhejiang and Fujian gradually receded northward [69], the water temperature rebounded, and the nutrient-rich warm currents of Taiwan were conducive to the reproduction of bait organisms [64]. At this time, a large number of nekton migrate to the sea area around the Ningde NPP for food and to reproduce. Therefore, the greatest number of nekton species occurred in summer and autumn. In winter, the cold air from the north and the cold water along the coast of Zhejiang and Fujian affect the water temperature and the food resources (e.g., plankton), both of which drop to the lowest level of the year. At this time, species that are sensitive to temperature change migrate to a lower latitude for overwintering [70]. This scenario explains why we found the lowest number of nekton species in winter.

### 4.2. Dominant Species of Nekton in the Study Area

The IRI analysis identified 32 species of nekton as dominant species over the 12 months of our study, with 19, 13, 14, and 13 dominant species in autumn, winter, spring, and summer, respectively. *O. oratoria*, *P. trituberculatus*, and *C. stigmatias* were dominant species in all four seasons. The dominant species are mainly small fish and invertebrates, and most of them live in the lower and middle layers of the sea. This distribution is related to the characteristics of the ecosystem of the near-shore sea area, where the near-shore runoff brings rich nutrients, providing a good habitat for nekton in the lower and middle layers of the sea.

We found that *O. oratoria* was the core dominant species in the sea area around the Ningde NPP, as it maintained dominance for 11 consecutive months, with IRI values that reached 7023 (Table 1), far exceeding those of other species [65,71]. As a perennial crustacean with strong nekton swimming ability, *O. oratoria* is known for its intense generalist feeding, often feeding on fish and shrimp [72]. Fish such as *C. lucidus*, *A. argentatus*, *H. nehereus*, *Odontamblyopus lacepedii*, and *T. uagina* and crabs such as *Eucrate crenata*, *Charybdis japonica*, and *P. trituberculatus*, which occur frequently and are native species, were also dominant in the study area [52]. The high frequency of occurrence of these native species indicates that they occupy an important position in the ecosystem of this marine area [52]. Kneib and Knowlton [73] found that the community structure of nekton in the eastern waters of the United States was dominated by seasonal migratory types and supplemented by local sedentary species. The dominant species investigated in the waters of the Qizing Islands was dominated by *P. trituberculatus* [65], as was true in our study. Changes in the biomass and abundance of these nekton species, which can occur as a result of seasonal and anthropogenic activities, can pose a risk to the safety of the cooling water system of the NPP.

### 4.3. Nekton Diversity in the Study Area

A more favorable biological environment results in a greater number of species, higher evenness, and lower prominence of the dominant species [74]. During the 12 months of our survey, *H* ranged from 1.80 to 2.84, with a mean value of 2.38. In the Taiwan Strait, Song et al. [75] found that the *H* of nekton ranged from 1.45 to 3.21, with a mean value of 2.47, *D* ranged from 1.78 to 2.98, with a mean value of 2.40, and *J* ranged from 0.65 to 0.87, with a mean value of 0.75. Desrita et al. [76] reported that the index of nekton diversity in Batang Toru River ranged from 1.20 to 2.40, and Song et al. [77] reported that it ranged from 1.40 to 2.60 in Fujian Sandu Bay.

Despite the differences in geographic location and fishing methods used in these various studies, nekton diversity in the Ningde NPP area was within the range of values for Sandu Bay, Fujian, and Toru River, Batang, indicating stable diversity and community structure. This moderate level can be explained by the rich nutrients and relatively stable habitat of the study area compared to the characteristics of the temperate and boreal zones. The study area is dominated by small fish and invertebrates, so the predation pressure on the nekton community is not great. Additionally, the number of nekton species is relatively large, with a uniform distribution of the species [78,79].

### 4.4. Spatial and Temporal Analysis of Nekton and Their Correlation with Environmental Factors

The nekton showed significant seasonal differences during the year-long study, with biomass and abundance values ranked as follows: summer > autumn > spring > winter. Nekton resources were relatively high from May to October and lower from November to April. The highest density of nekton occurred in June (summer), with mean biomass and mean abundance of 399.53 kg/km^2^ and 61.98 × 10^3^ ind/km^2^, respectively. The lowest density occurred in January (winter), with mean biomass and mean abundance of 96.82 kg/km^2^ and 7.15 × 10^3^ ind/km^2^, respectively. From May to October, the biomass and abundance of nekton were higher than the monthly mean values of 194.29 kg/km^2^ and 25.34 × 10^3^ ind/km^2^. The values in June were 2.05 and 2.45 times higher than the monthly mean values, respectively, 4.13 and 8.66 times higher than those in January, and 3.25 and 2.82 times higher than those in May. This indicates that the density of nekton fluctuates in different months, with significant interannual differences. Factors that can influence the abundance and biomass of nekton include sea surface temperature, salinity, season, and habitat [80,81,82,83].

Taxonomically, mean monthly fish biomass and abundance (84.01 kg/km^2^ and 12.71 × 10^3^ ind/km^2^) were significantly higher than those of shrimp (53.90 kg/km^2^ and 9.02 × 10^3^ ind/km^2^) and crabs (53.09 kg/km^2^ and 3.26 × 10^3^ ind/km^2^) in the study area. Nekton abundance increased significantly in summer from June onwards. The biomass and abundance of fish contributed more than 68.75% to the total nekton values, suggesting that fish began to congregate in large numbers beginning in June. This scenario can be explained by the migration of small fish and invertebrates to the area to spawn in the spring, which results in a high number of larvae but small biomass [61]. The average biomass in summer and autumn is higher than that in spring, likely because juvenile fish gradually develop into adults and/or seasonal environmental factors such as water temperature, light, and food supply lead to increased growth [81,82]. In winter, some fish and crustaceans migrate to lower latitudes due to factors such as water temperature and food supply [83], which results in decreased biomass and abundance. There is a certain correlation between the species composition and quantitative trends of nekton and plankton. Zhang et al. [43] reported that in the Tiaowei Island sea area, the maximum number of zooplankton species reached 80 in summer, with *Oithona copepods* being the most abundant group. In another study by Zhang et al. [44] near the Ningde NPP, the density of phytoplankton peaked in autumn at 2.90 × 10^4^ cells/L, while the density of zooplankton was highest in summer at 400.63 ind/m^3^. This study found that the species number, abundance, and biomass of nekton were all highest in summer. The annual pattern of change in nekton abundance suggests that May–October is the peak period for nekton abundance, and during this period, nekton may pose a risk to the cooling water system in the NPP power intake area.

The distribution of nekton is closely related to the marine environment, food availability, migration, and seafloor geomorphology [18,77,83]. The survey showed that there was a certain spatial variability in the abundance and biomass of nekton, and the abundance and biomass of the stations (e.g., 4# and 8#) within the 10 km line from the cooling water system were higher than those stations near the 20 km line and 30 km line (Figure 6), indicating that the remote stations had little influence on the cooling water system of the nuclear power plant. According to RDA and person analysis results, the abundance and biomass of nekton were mainly affected by pH, DIP, and DIN. Changes in pH affect the structure and abundance of nekton communities, thus the process of ocean acidification adversely affects the physiological processes of nekton [84]. Our results showed a significant negative correlation between pH and the amount of nekton. DIP and DIN also affect the nekton community structure and abundance by influencing the base of the food chain [43,85]. We detected a positive correlation between DIP and DIN levels and the amount of nekton present in the study area. Zhang et al. [86] also found that pH, DIP, and DIN were the main environmental factors affecting the nekton community in the eastern waters of Laizhou Bay.

Salinity, water temperature, turbidity, DO content, freshwater runoff, and habitat characteristics are also important factors that affect the spatial and temporal distribution patterns of nekton [43,85,87]. Song et al. [53] used RDA to assess the community structure of nekton in the Oujiang Estuary in Zhejiang Province and found that it was mainly driven by DIN.

### 4.5. Potential Risks of Nekton to the Safety of the Cooling Water System of the Ningde NPP

The generation of thermal wastewater from coastal power plants is known to affect marine organisms [88,89]. With climate-induced changes in the marine environment, the influence of marine organisms on the safe operation of the cooling water system of coastal NPPs has become a research hotspot. However, little information related to nekton in the sea area around the Ningde NPP was available before our study.

Lin et al. [5] and Dai et al. [90] found that the cooling source of the coastal power plant was vulnerable to the impacts of small schooling fish and nekton in the spring and summer seasons. Impacts of aggregations of juveniles with weaker swimming ability, such as *H. nehereus*, anchovy (*Stolephorus* sp.), and *A. argentatus* are of concern. Tang et al. [91] reported that the cold source of the Ningde NPP was invaded by a large number of *O. oratoria* in the autumn of 2016, which led to the power reduction in the unit. These results indicate that pelagic nekton, such as juvenile and young fish, as well as *O. oratoria* and other bottom nekton pose risks to the intake of the NPP in spring and summer. We found that the body length of *O. oratoria* ranged from 2.5 cm to 14 cm, with an average length of 5 cm, and that most of them were juveniles inhabiting the muddy seabed. They move easily with the water flowing into the intake area during periods of extreme weather. The dominant species in our study differed slightly from those reported by Dai et al. [90], and our biomass and abundance values were higher than those from their survey. These differences may be due to different survey methods, times, and sea areas. Future studies should explore the effects of these potential factors on the community structure of nekton to gain a more comprehensive understanding of the ecosystem characteristics of this sea area.

Researchers use many different screening criteria depending on the group of organisms that clog the cooling water system of coastal NPPs. Wang et al. [33] screened phytoplankton species for potential risk based on cell diameter, outbreak or aggregation events, and species abundance. Zhang et al. [44] used domestic and international reported species, the morphology of the causal organisms, the similarity of habits, and the aggregation events as the criteria for their classification. In other studies, researchers used criteria such as marine biota exceeding the mesh diameter (3 mm) of the intake drum mesh of an NPP, those with weak motility, and those prone to aggregation or outbreaks to identify species that may pose a threat to the cooling water system of an NPP [8,92]. Using hierarchical analysis, Tang [13] proposed for the first time the principle of screening organisms for their risk of blocking the intake of the Ningde NPP. They found that jellyfish are risk organisms, and they created a screening index system.

In this study, we used the proportion of juveniles in the total nekton > 30%, a large number of aggregation events occurring in the surrounding waters, and nekton biomass > 5.00 kg/km^2^ and abundance > 20.00 × 10^3^ ind/km^2^ as criteria to screen for potentially risky nekton species (Table 3). Four nekton species satisfied these three criteria at the same time: *C. lucidus*, *H. nehereus*, *A. argentatus*, and *O. oratoria* (Table 3). The proportion of larvae in the nekton and the abundance of nekton are important references, and more attention should be paid to the months with a high proportion of larvae and high catch resources. Most fish in the waters of the East China Sea enter the breeding season in spring [93], and reproduction produces a large number of larvae. We found that the proportion of nekton larvae was highest in May in the Ningde NPP area, and the proportion of *C. lucidus*, *H. nehereus*, and *A. argentatus* larvae exceeded 30%. The biomass and abundance of *H. nehereus* and *A. argentatus* increased beginning in May and peaked in the summer, so their risk to the water cooling system of the NPP was highest in spring and summer. Similarly, Deng et al. [42] used abundance to screen the nekton catch and found that jellyfish were most abundant in spring and summer, thereby posing a high risk of clogging the cold water intake source of the Ningde NPP.

In our study, the nekton biomass and abundance were highest from May to October, and this could lead to the aggregation of a large number of marine organisms in the cooling water intake system if abnormal weather events occur [5]. Therefore, we propose that the Ningde NPP strengthen its pre-warning, monitoring, biological prevention, control, and extermination work to reduce the risk of excessive aggregation of nekton in the intake area during this period. Improving the pre-warning and monitoring systems and installing additional blocking nets should be prioritized [9,94]. Research on disperse and extermination technology to minimize the potential threat of Marine organisms to the safe operation of the cooling water system [44].

## 5. Conclusions

In this study, we assessed the community structure of nekton and its correlation with environmental factors in the sea adjacent to Ningde NPP through a one-year systematic survey. We recorded 120 species of nekton in 20 orders, 57 families, and 92 genera. *O. oratoria*, *P. trituberculatus*, and *C. stigmatias* were dominant species in all four seasons. pH, PIN, and DIN were the main environmental factors affecting nekton community structure in spring and autumn. Analysis of dominant species, abundance and biomass, and risk calendars revealed that May–October is a high-risk period for nekton to affect the safe operation of the cooling water system of the coastal NPP. Attention should be paid to species that present a potential risk, such as *C. lucidus*, *H. nehereus*, *A. argentatus*, and *O. oratoria*. We propose that the Ningde Nuclear Power Plant (NPP) strengthen its pre-warning, monitoring, prevention, control, and elimination of marine organisms to reduce the risk of excessive gathering of nekton in the intake area during high-risk periods. Monitoring nekton and research in this area is of practical significance to ensure the safe operation of the nuclear power cooling source. Additionally, the diversity and abundance data provide a theoretical basis for biological and ecological restoration and management in the area around the Ningde NPP.

## Figures and Tables

**Table 1:** Seasonal variation in the index of relative importance (IRI) of dominant nekton species.

Species Name	September 2022	October 2022	November 2022	December 2022	January 2023	February 2023	March 2023	April 2023	May 2023	June 2023	July 2023	August 2023
*Loligo beka*	127	131								675	42	123
*Oratosquilla oratoria*	2112	6246	6939	484	7023	2276	2012	2805	1944	1298	3567	2022
*Parapenaeopsis hardwickii*	539	1364	29	55	14	6		142	335	656	1510	2799
*Alpheus japonicus*	8		57		1923	4226	4106	1905	634	68	32	81
*Exopalaemon carinicauda*			27	344	425	642	585	370	45	348	46	41
*Oratosquilla interrupta*	20	838	845	10	18				13	33	16	
*Dictyosquilla foveolata*	443	851	112	22				66	337		345	188
*Parapenaeopsis tenella*			558	140		15	5	62	1370			
*Solenocera crassicornis*	1048	180	726				5				465	
*Erugosquilla woodmasoni*		828	81									
*Eucrate crenata*	392	175	357	940	492	391	1505	1777	607	772	1226	119
*Charybdis japonica*	1066	793	732	1420	263	223	29	469	349	126	24	175
*Portunus trituberculatus*	1406	1284	1673	3206	259	165	79	1251	1988	3795	2923	1219
*Portunus hastatoides*	15	43	35	42				148	131	885	21	18
*Charybdis bimaculata*	43		488	36	13			46	971	332		8
*Portunus sanguinolentus*	462	1069								2	552	255
*Portunus pelagicus*	1009	768	1529	128	73							
*Odontamblyopus rubicundus*	234	34	414	1974	519	75	493	400	647	219	762	240
*Trypanuchen uagina*	49	54	990	1796	598	385	2387	4750	1141	240	496	41
*Cynoglossus semilaevis*	30			15	468	156	702	85	39	84	29	60
*Johnius belangerii*			126	237	171	501	409	36	57	8	29	6
*Chaeturichthys stigmatias*			1887	4344	457	1837	2285	207	9	776	5	302
*Chaeturichthys hexanema*	76	585	834	1106	162	1466	1917	1300	3018	372		
*Harpodou nehereus*	24	1424	177	16				89	1326	300	402	2328
*Collichthys lucidus*	6	379	128	487	5222	6347	2308	1204	581			
*Chrysochir aureus*	40	191	572	718	824	361	23			55		71
*Argyrosomus argentatus*	184	238	10		116				34	5001	3310	3822
*Ilisha elongata*	4234	867	7	13								145
*Thrissa mystax*	2908	792	37			8		313				
*Polydactylus sextarius*	6	48									1212	474
*Leiognathus ruconius*	2259										10	6
*Saurida elongata*	9										552	

**Table 2:** Comparison of results of historical surveys of neighboring seas.

Year	Seawater	Nekton Community Structure	Survey Season	Network Mesh Size (cm)	Trawl Time (h)
2008 [65]	Sansha Bay	Fish 94 species	Spring and Autumn	1.7	0.33
2012–2013 [66]	Taishan Islands	Nekton 136 species, Fish 80 species, Crustaceans 44 species, Cephalopods 9 species	Spring, Summer, Autumn and Winter	1.3–2.5	0.5–1.0
2014–2015 [63]	Qixing Islands	Nekton 80 species, Fish 52 species, Crustaceans 23 species, Cephalopods 5 species	Spring and Autumn	2.0	0.5
2016 [64]	Minjiang kou River	Fish 125 species, 13 orders	Spring, Summer, Autumn and Winter	2.0	0.5
2020 [52]	Qingchuan Bay	Fish 55 species 11 orders 29 families 49 genera	Spring and autumn	2.0	0.5
2022–2023 (This study)	Qingchuan Bay	120 species of nekton, 72 species of fish, 23 species of crustaceans, 5 species of cephalopods	Spring, Summer, Autumn and Winter	2.0	0.5
2023 year [53]	Oujiang River Estuary	78 species of nekton, 36 species of fish, 28 species of crustaceans, 3 species of cephalopods	Spring and Autumn	2.0	0.5–1.0

**Table 3:** Thirty-two nekton species pose a potential risk to the Ningde NPP and a monthly calendar of risks to the NPP based on screening criteria.

Species	Jan	Feb	Mar	Apr	May	Jun	Jul	Aug	Sep	Oct	Nov	Dec	Proportion of Larvae (%)	Min Biomass (kg/km^2^)	Max Biomass (kg/km^2^)	Min Abundance (×10^3^ ind./km^2^)	Maximum Abundance (×10^3^ ind./km^2^)	Comprehensive Risk Level
*Argyrosomus argentatus*													50	3.34	22.08	22.90	58.27	***
*Cynoglossus semilaevis*													5	0.31	0.31	5.19	5.19	*
*Oratosquilla interrupta*													5	0.62	1.55	3.38	13.21	*
*Parapenaeopsis hardwickii*													10	1.60	9.40	5.05	35.07	**
*Portunus sanguinolentus*													0	0.57	1.43	3.71	22.78	*
*Loligo beka*													50	1.69	1.69	16.27	16.27	*
*Collichthys lucidus*													35	0.26	5.78	5.70	67.50	**
*Exopalaemon carinicauda*													5	0.52	0.69	2.48	3.35	*
*Chrysochir aureus*													40	0.23	0.54	2.23	4.91	*
*Trypanuchen uagina*													35	0.23	2.45	2.70	26.66	*
*Oratosquilla oratoria*													35	1.45	10.46	10.96	110.59	***
*Odontamblyopus rubicundus*													10	0.31	1.31	0.87	7.76	*
*Ilisha elongata*													5	1.40	10.34	15.90	55.69	*
*Chaeturichthys hexanema*													25	0.68	5.91	2.72	6.86	*
*Polydactylus sextarius*													0	1.38	1.38	6.85	6.85	*
*Harpodou nehereus*													35	0.69	5.71	12.45	42.39	***
*Eucrate crenata*													5	0.32	1.75	4.50	19.79	**
*Leiognathus ruconius*													0	7.82	7.82	13.29	13.29	*
*Chaeturichthys stigmatias*													25	1.26	4.17	4.18	18.12	**
*Portunus hastatoides*													0	5.00	5.00	3.18	3.18	*
*Johnius belangerii*													35	0.48	0.48	3.15	3.15	*
*Alpheus japonicus*													5	1.09	6.32	1.35	9.10	*
*Charybdis japonica*													0	0.25	1.34	5.44	23.06	*
*Portunus trituberculatus*													0	0.54	2.60	8.00	136.63	**
*Charybdis bimaculata*													0	1.66	1.66	2.64	2.64	*
*Dictyosquilla foveolata*													5	1.18	1.18	17.65	17.65	*
*Erugosquilla woodmasoni*													5	1.26	1.26	15.99	15.99	*
*Parapenaeopsis tenella*													5	0.60	2.34	0.53	3.76	*
*Portunus pelagicus*													0	0.32	1.22	13.04	22.19	*
*Saurida elongata*													0	0.32	0.32	6.02	6.02	*
*Thrissa mystax*													0	1.83	7.57	8.63	34.90	*
*Solenocera crassicornis*													5	0.65	6.52	1.87	22.78	*
	low	medium	high	extreme high														

Note: The symbol * represents the blockage risk level from nekton species. * indicates a low comprehensive risk, ** indicates a moderate risk, and *** indicates a high risk.

## Data Availability

The original contributions presented in this study are included in the article/Supplementary Material. Further inquiries can be directed to the corresponding author(s).

## Supplementary Materials

The following supporting information can be downloaded at: https://www.mdpi.com/article/10.3390/biology14050481/s1. Table S1. Normality tests for diversity indexes: Shannon (*H*), Pielou_e (*J*), Margalef (*D*); Figure S1. Normal Q-Q plots of Shannon index (*H*) for the autumn group; Figure S2. Normal Q-Q plots of Shannon index (*H*) for the winter group; Figure S3. Normal Q-Q plots of Shannon index (*H*) for the spring group; Figure S4. Normal Q-Q plots of Shannon index (*H*) for the summer group; Figure S5. Normal Q-Q plots of Pielou_e index (*J*) for the autumn group; Figure S6. Normal Q-Q plots of Pielou_e index (*J*) for the winter group; Figure S7. Normal Q-Q plots of Pielou_e index (*J*) for the spring group; Figure S8. Normal Q-Q plots of Pielou_e index (*J*) for the summer group; Figure S9. Normal Q-Q plots of Margalef index (*D*) for the autumn group; Figure S10. Normal Q-Q plots of Margalef index (*D*) for the winter group; Figure S11. Normal Q-Q plots of Margalef index (*D*) for the spring group; Figure S12. Normal Q-Q plots of Margalef index (*D*) for the summer group; Table S2. Descriptive statistics of Shannon index (*H*); Table S3. Homogeneity test for Shannon index (*H*); Table S4. Descriptive statistics of Pielou_e index (*J*); Table S5. Homogeneity test for Pielou_e index (*J*); Table S6. Descriptive statistics of Margalef index (*D*); Table S7. Homogeneity test for Margalef index (*D*); Table S8. Normality test for environmental and nekton variables in autumn and spring; Figure S13. Normal Q-Q plots of temp for the autumn group; Figure S14. Normal Q-Q plots of temp for the spring group; Figure S15. Normal Q-Q plots of sal for the autumn group; Figure S16. Normal Q-Q plots of sal for the spring group; Figure S17. Normal Q-Q plots of pH for the autumn group; Figure S18. Normal Q-Q plots of pH for the spring group; Figure S19. Normal Q-Q plots of DO for the autumn group; Figure S20. Normal Q-Q plots of DO for the spring group; Figure S21. Normal Q-Q plots of COD for the autumn group; Figure S22. Normal Q-Q plots of COD for the spring group; Figure S23. Normal Q-Q plots of DIP for the autumn group; Figure S24. Normal Q-Q plots of DIP for the spring group; Figure S25. Normal Q-Q plots of DIN for the autumn group; Figure S26. Normal Q-Q plots of DIN for the spring group; Figure S27. Normal Q-Q plots of Si for the autumn group; Figure S28. Normal Q-Q plots of Si for the spring group; Figure S29. Normal Q-Q plots of DEEP for the autumn group; Figure S30. Normal Q-Q plots of DEEP for the spring group; Figure S31. Normal Q-Q plots of biomass for the autumn group; Figure S32. Normal Q-Q plots of biomass for the spring group; Figure S33. Normal Q-Q plots of abundance for the autumn group; Figure S34. Normal Q-Q plots of abundance for the spring group; Figure S35. Normal Q-Q plots of C for the autumn group; Figure S36. Normal Q-Q plots of C for the spring group; Figure S37. Normal Q-Q plots of Shannon index (*H*) for the autumn group; Figure S38. Normal Q-Q plots of Shannon index (*H*) for the spring group; Figure S39; Normal Q-Q plots of Pielou_e index (*J*) for the autumn group; Figure S40. Normal Q-Q plots of Pielou_e index (*J*) for the spring group; Figure S41. Normal Q-Q plots of Margalef index (*D*) for the autumn group; Figure S42. Normal Q-Q plots of Margalef index (*D*) for the spring group; Table S9. Homogeneity test for environmental and nekton variables in autumn and spring; Table S10. Nested ANOVA of Shannon index (*H*); Table S11. Multiple comparisons for Shannon index (*H*); Figure S43. Standardized residual plot of observed vs. predicted Shannon index (*H*); Table S12. Nested ANOVA of Pielou_e index (*J*); Table S13. Multiple comparisons for Pielou_e index (*J*); Figure S44. Standardized residual plot of observed vs. predicted Pielou_e index (*J*); Table S14. Nested ANOVA of Margalef index (*D*); Table S15. Multiple comparisons for Margalef index (*D*); Figure S45. Standardized residual plot of observed vs. predicted Margalef index (*D*).
