# Supplementary material for: Community Characteristics and Potential Risk of Nekton in Waters Adjacent to Ningde Nuclear Power Plant in Fujian, China
Source: Biology (Basel). 2025 Apr 27;14(5):481. doi: 10.3390/biology14050481 (PMC12108784; doi:10.3390/biology14050481)
Supplement: Supplementary file 1 [file biology-14-00481-s001.zip › biology-3425836-supplementary.pdf]

SUPPLEMENTARY MATERIAL

Table S1. Normality tests for diversity indexes: Shannon (*H*), Pielou_e (*J*), Margalef (*D*).

Tests of Normality							
	group	Kolmogorov-Smirnov[a]			Shapiro-Wilk		
		Statistical data	df	significance	Statistical data	df	significance
Shannon (*H*)	aut	.194	6	.200*	.886	6	.297
	win	.262	6	.200*	.884	6	.288
	spr	.213	6	.200*	.947	6	.718
	sum	.152	6	.200*	.960	6	.822
Pielou_e (*J*)	aut	.239	6	.200*	.960	6	.820
	win	.233	6	.200*	.908	6	.421
	spr	.253	6	.200*	.897	6	.359
	sum	.214	6	.200*	.842	6	.136
Margalef (*D*)	aut	.144	6	.200*	.989	6	.986
	win	.245	6	.200*	.852	6	.162
	spr	.156	6	.200*	.936	6	.627
	sum	.238	6	.200*	.898	6	.360
*. This is the significant lower bound of true.							
a. Lilliefors Significance Correction							

Note:

Season abbreviations: aut = autumn, win = winter, spr = spring, sum = summer.

Indexes: Shannon (*H*) = Shannon–Wiener's diversity index (*H*);

Pielou_e (*J*) = Pielou's evenness index (*J*);

Margalef (*D*) = Margalef's species richness index (*D*).

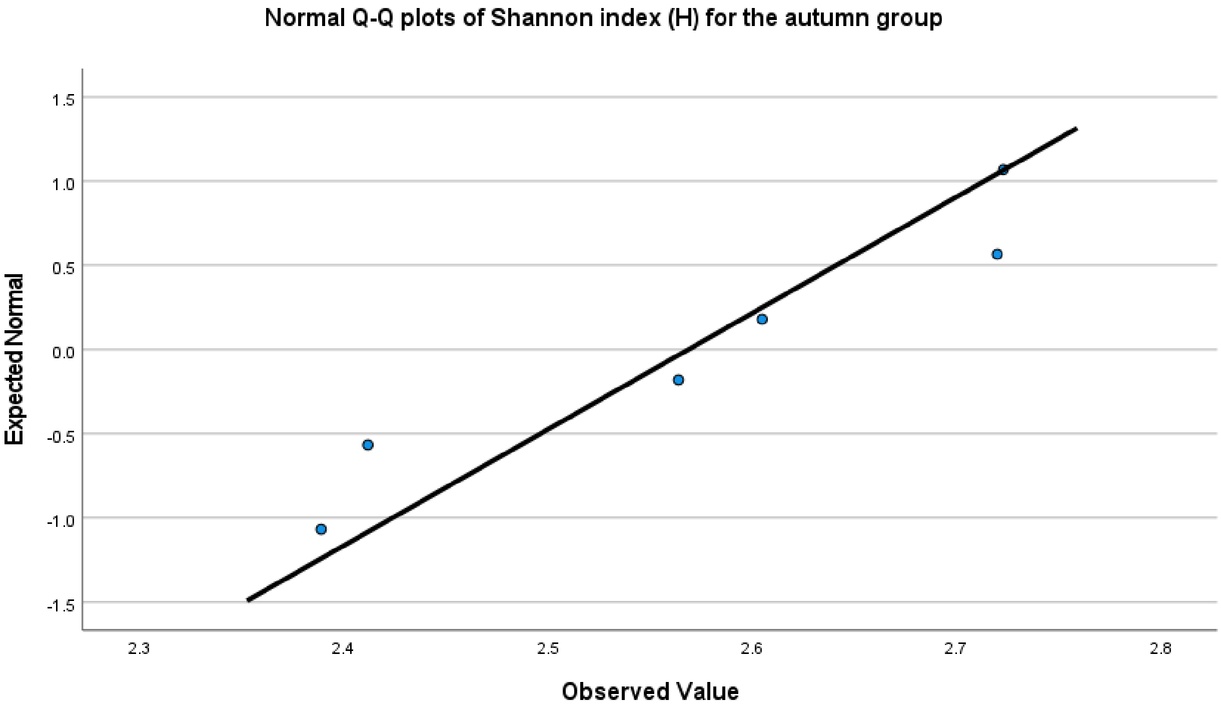

Figure S1. Normal Q-Q plots of Shannon index (*H*) for the autumn group.

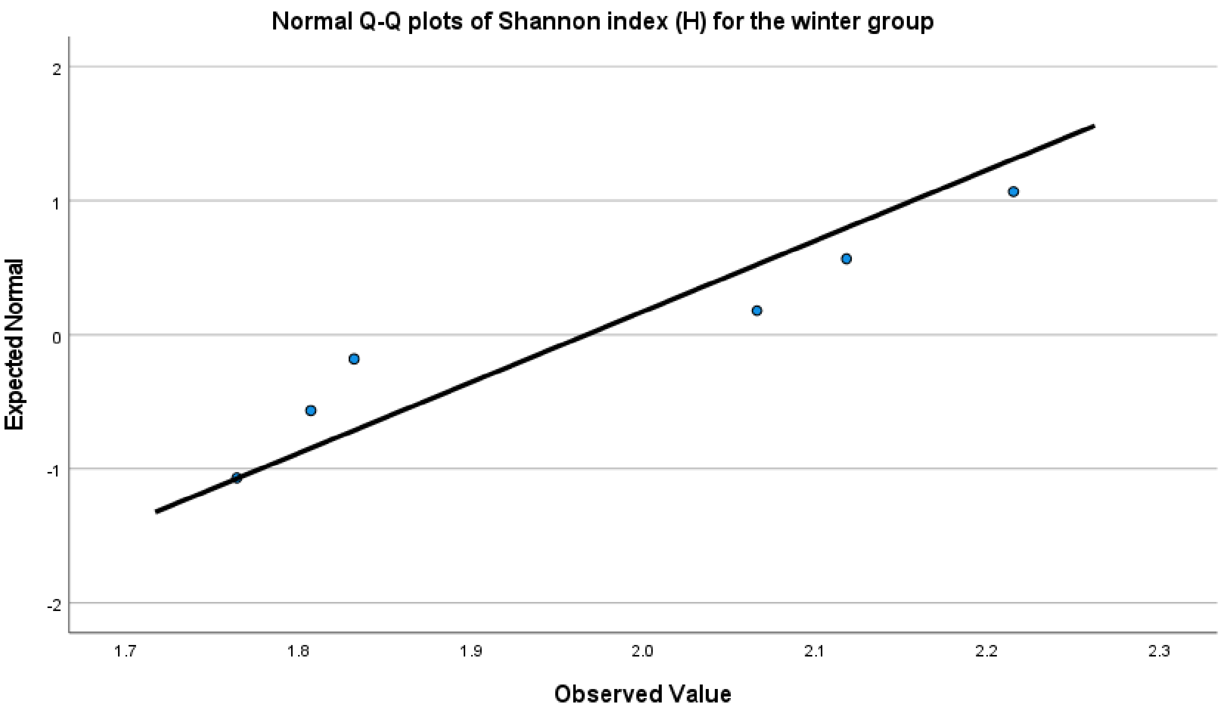

Figure S2. Normal Q-Q plots of Shannon index (*H*) for the winter group.

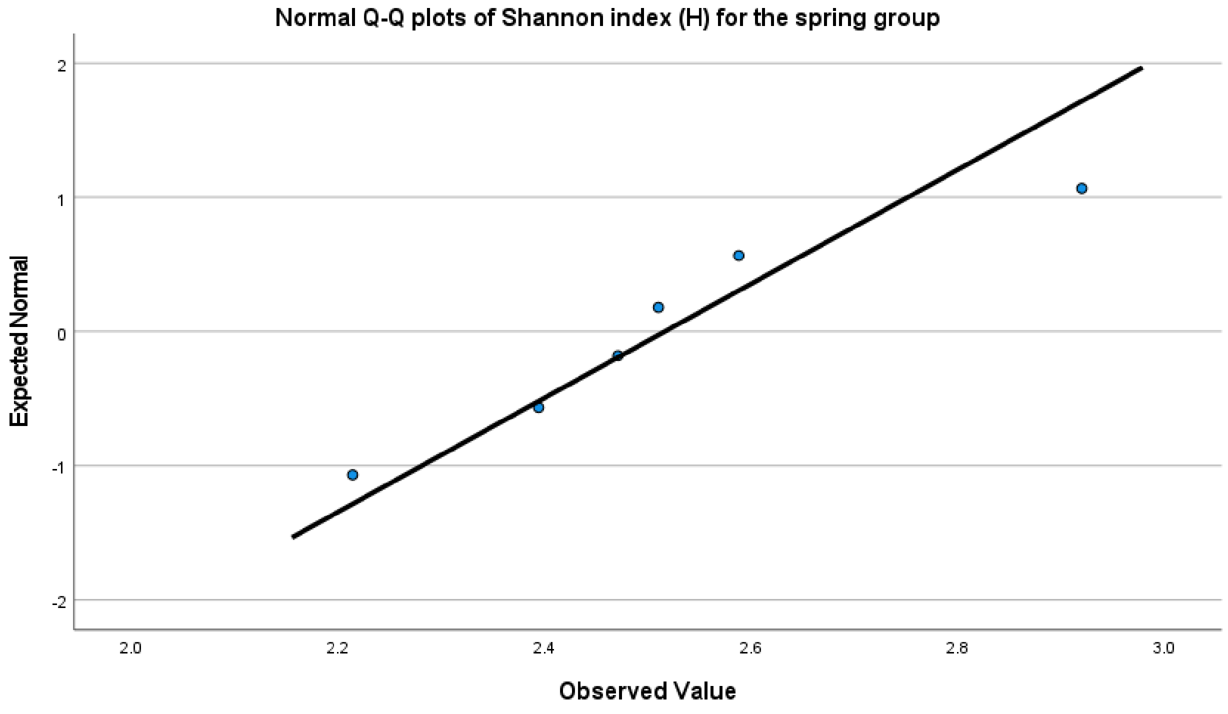

Figure S3. Normal Q-Q plots of Shannon index (*H*) for the spring group.

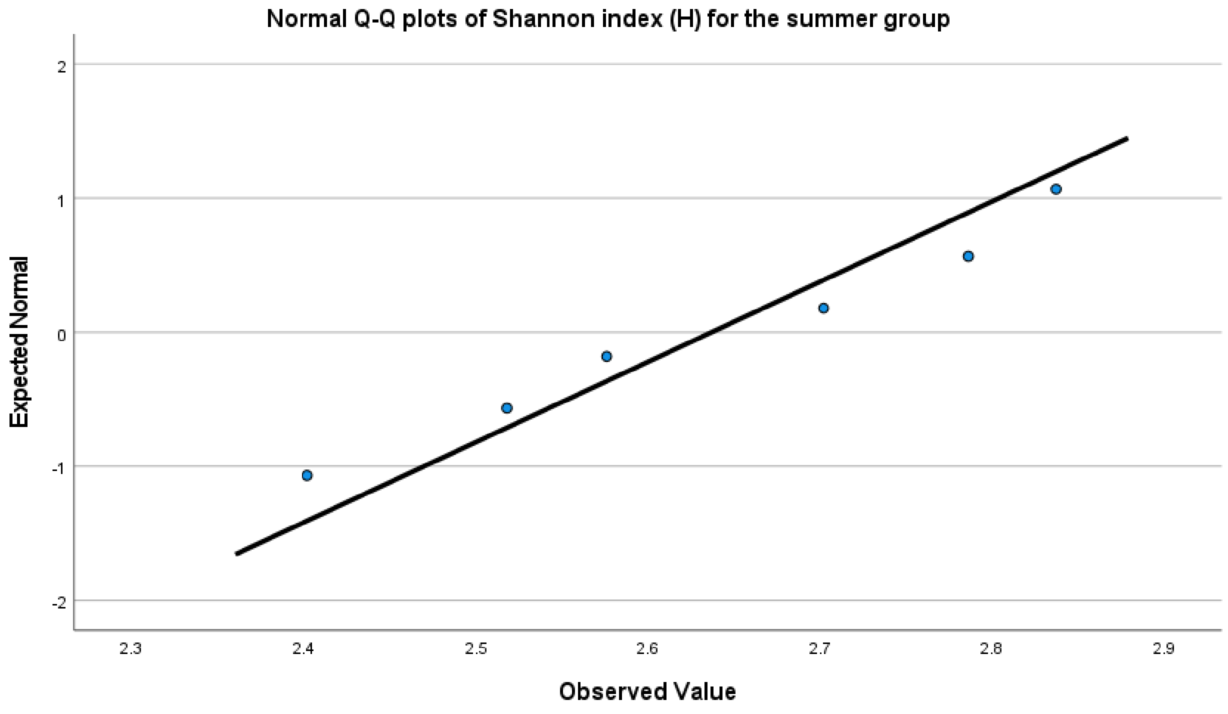

Figure S4. Normal Q-Q plots of Shannon index (*H*) for the summer group.

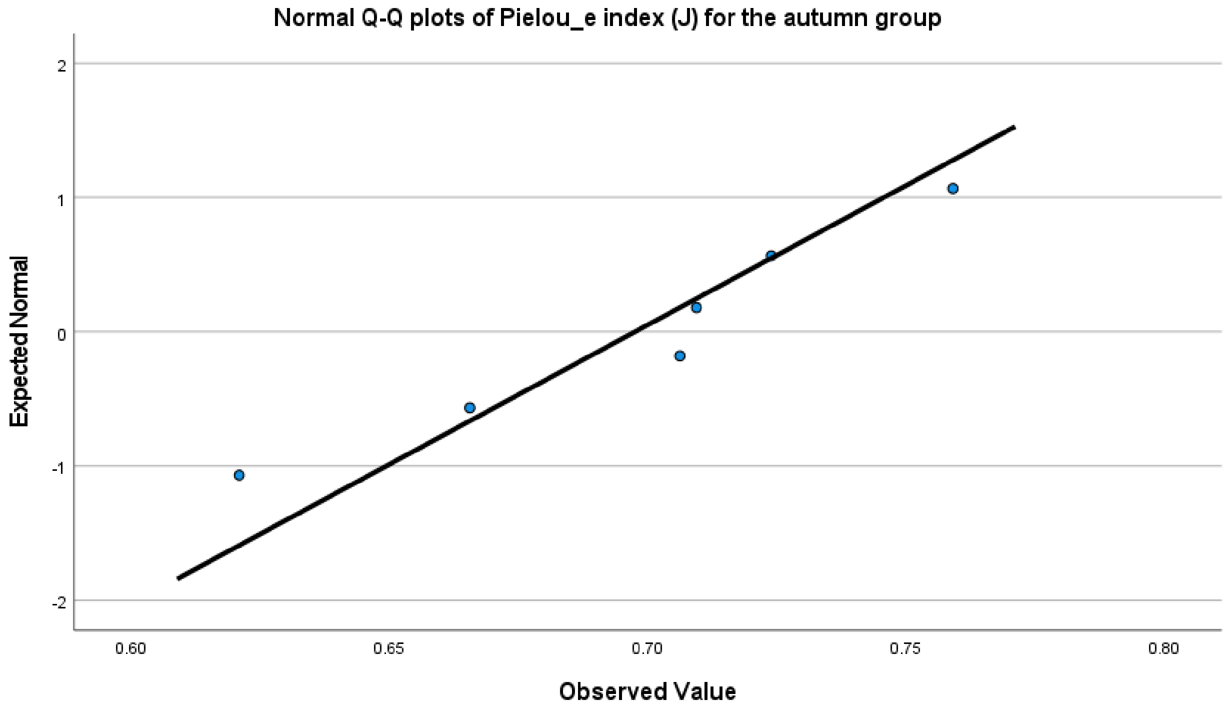

Figure S5. Normal Q-Q plots of Pielou_e index (*J*) for the autumn group.

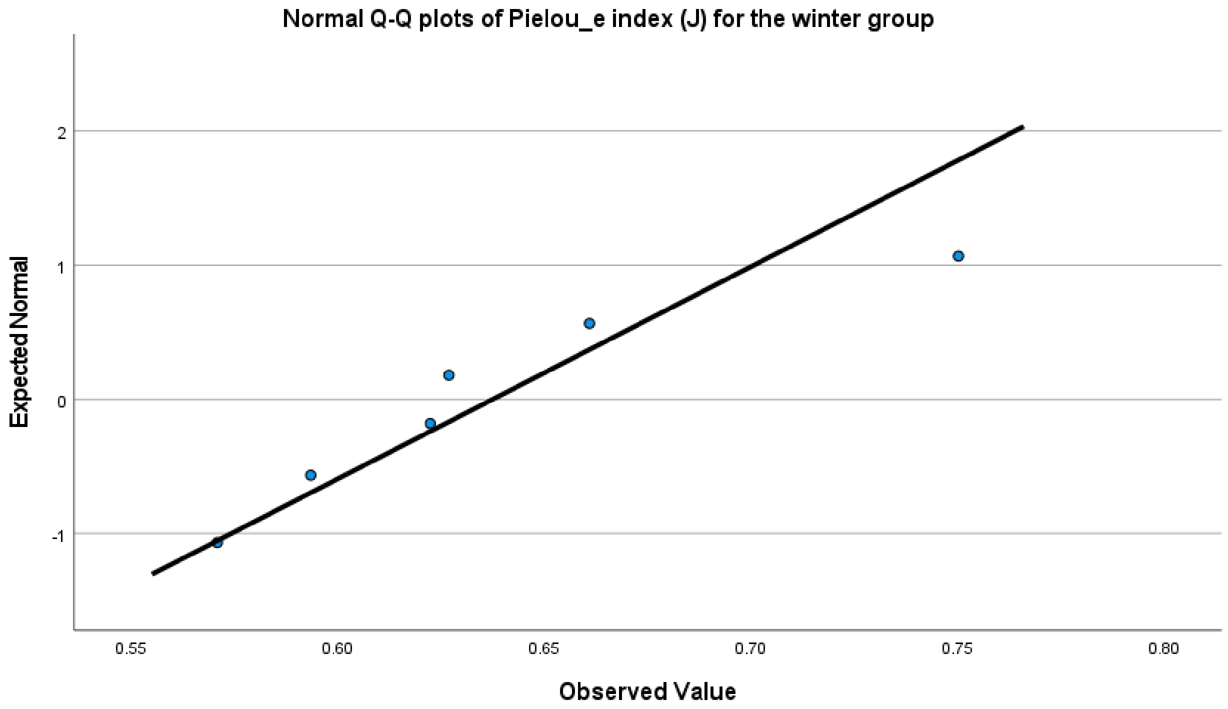

Figure S6. Normal Q-Q plots of Pielou_e index (*J*) for the winter group.

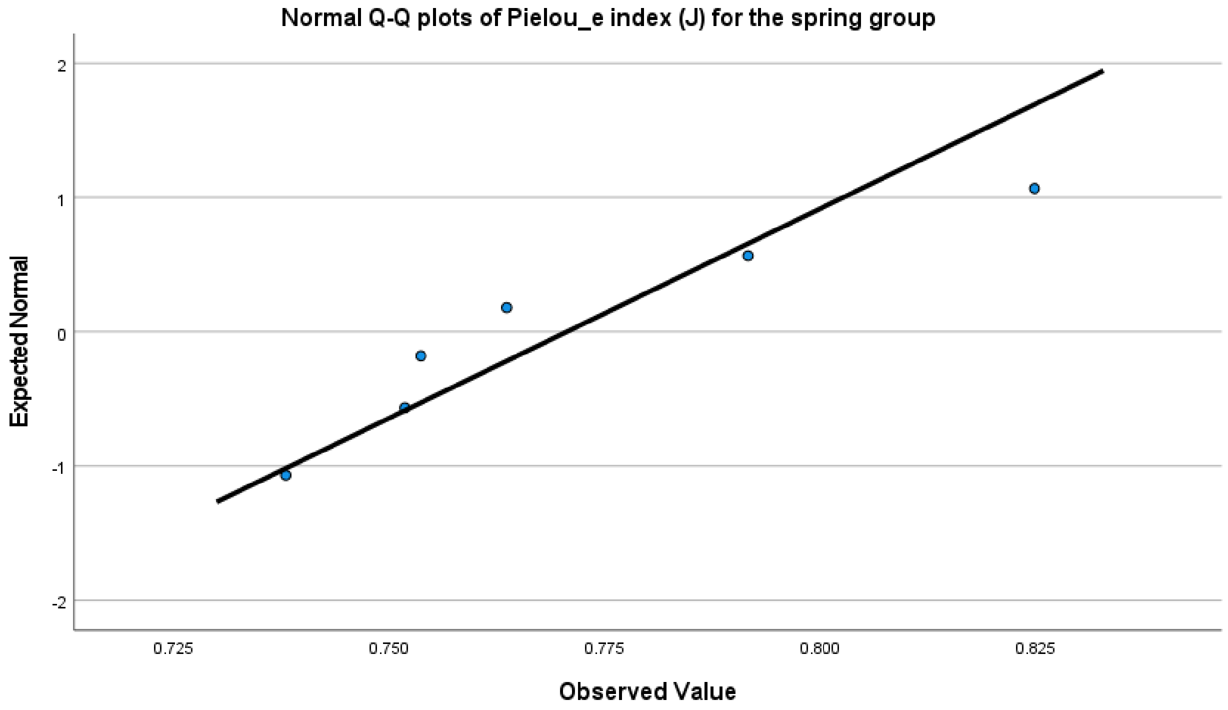

Figure S7. Normal Q-Q plots of Pielou_e index (*J*) for the spring group.

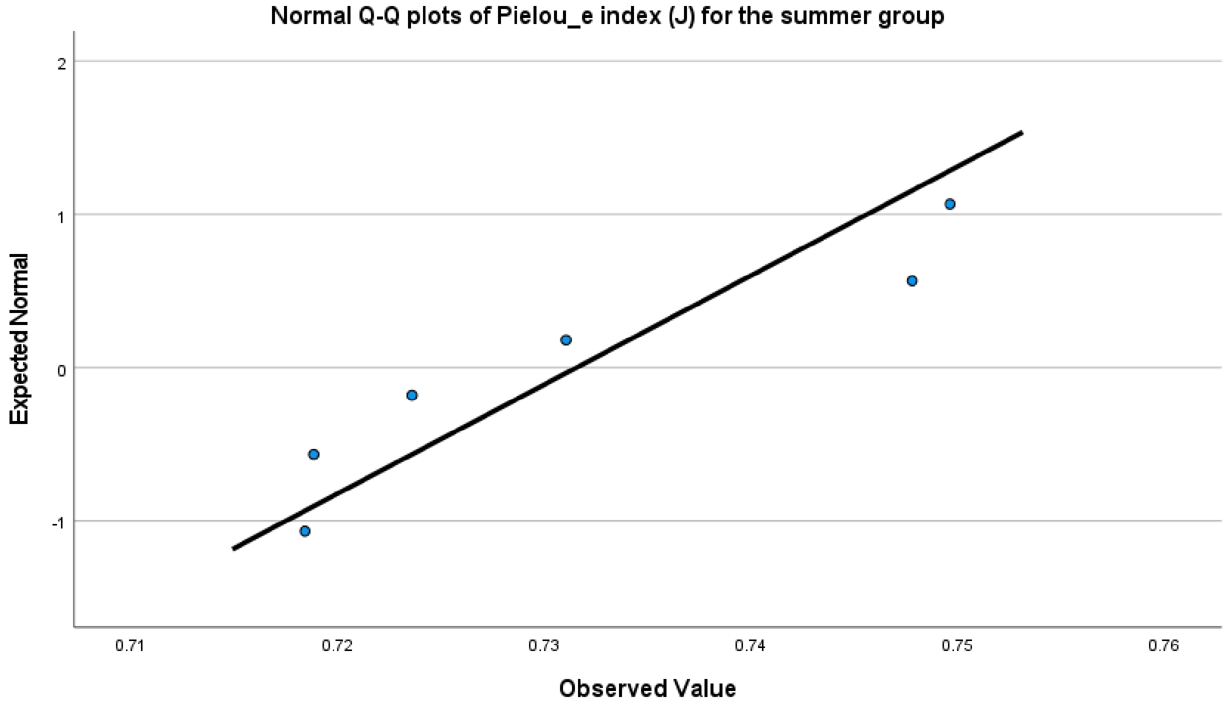

Figure S8. Normal Q-Q plots of Pielou_e index (*J*) for the summer group.

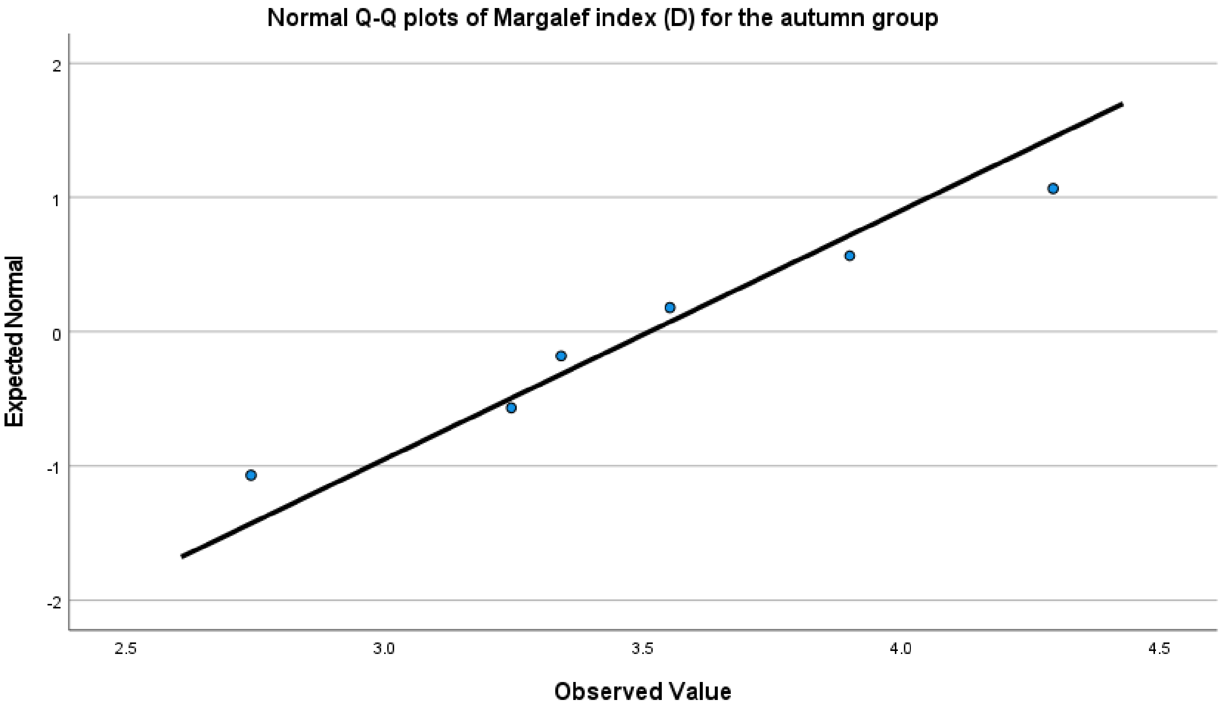

Figure S9. Normal Q-Q plots of Margalef index (*D*) for the autumn group.

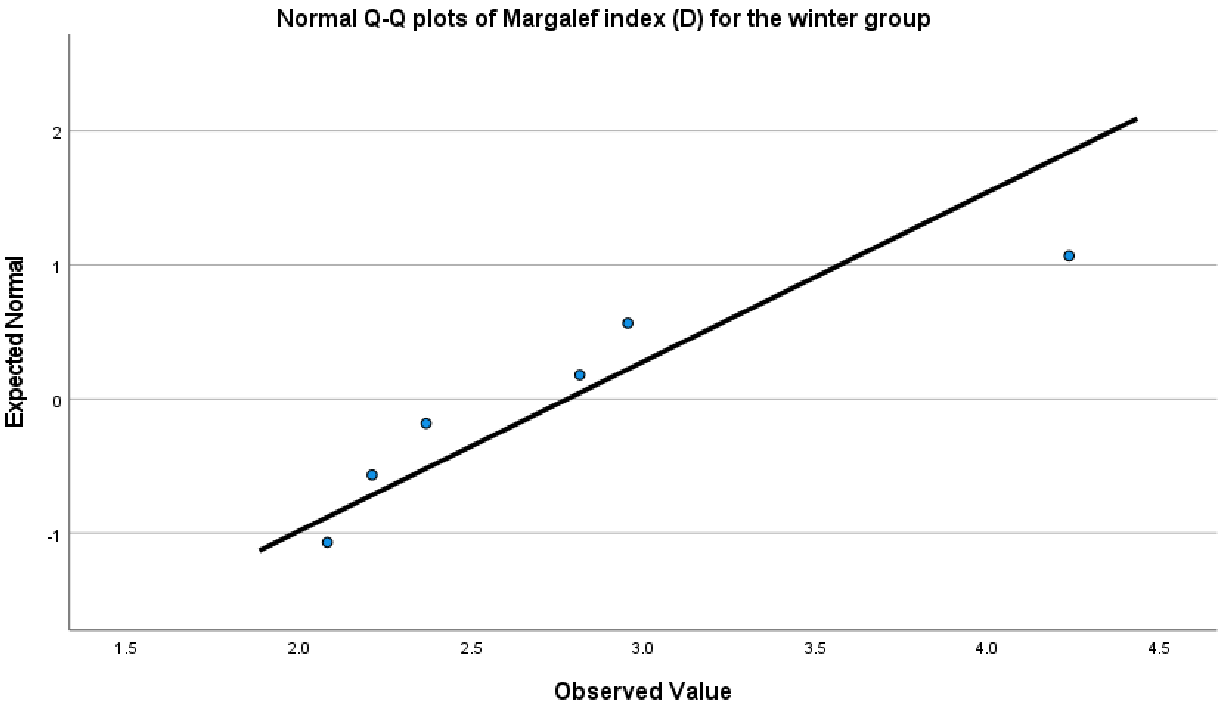

Figure S10. Normal Q-Q plots of Margalef index (*D*) for the winter group.

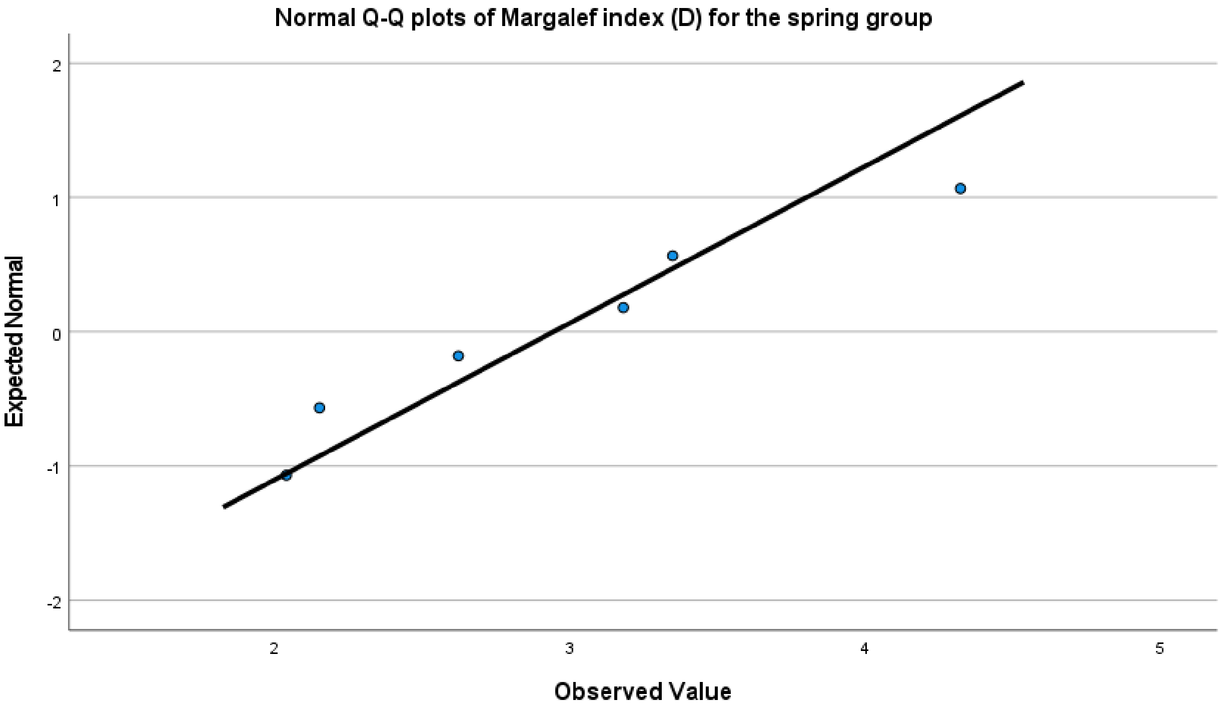

Figure S11. Normal Q-Q plots of Margalef index (*D*) for the spring group.

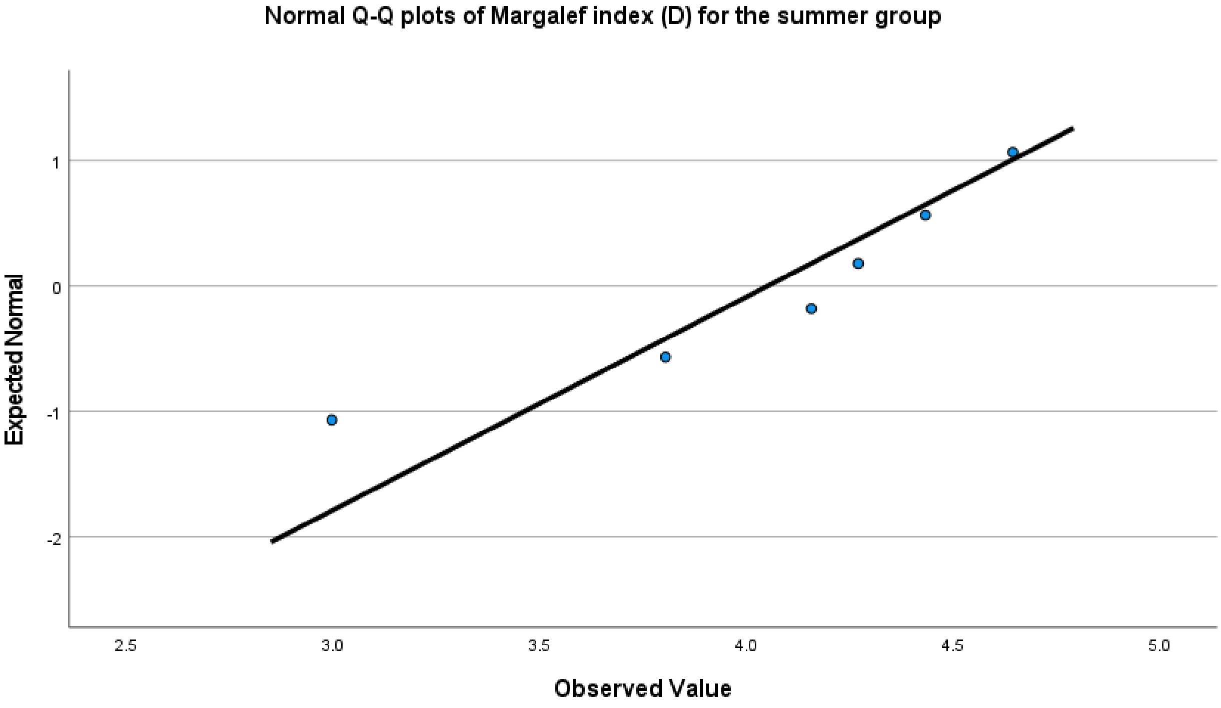

Figure S12. Normal Q-Q plots of Margalef index (*D*) for the summer group.

Table S2. Descriptive statistics of Shannon index (*H*).

Descriptive Statistics				
Dependent Variable: Shannon (*H*)				
Season	Month	Mean	Std. Deviation	N
aut	Sep	2.6640	.08344	2
	Oct	2.5545	.23405	2
	Nov	2.4880	.10748	2
	Total	2.5688	.14484	6
win	Jan	1.7980	.04808	2
	Feb	1.9365	.18314	2
	Dec	2.1665	.06859	2
	Total	1.9670	.18928	6
spr	Mar	2.3425	.18173	2
	Apr	2.4520	.08202	2
	May	2.7540	.23476	2
	Total	2.5162	.23518	6
sum	Jun	2.5520	.21213	2
	Jul	2.5470	.04101	2
	Aug	2.8115	.03606	2
	Total	2.6368	.16705	6
Total	Jan	1.7980	.04808	2
	Feb	1.9365	.18314	2
	Mar	2.3425	.18173	2
	Apr	2.4520	.08202	2
	May	2.7540	.23476	2
	Jun	2.5520	.21213	2
	Jul	2.5470	.04101	2
	Aug	2.8115	.03606	2
	Sep	2.6640	.08344	2
	Oct	2.5545	.23405	2
	Nov	2.4880	.10748	2
	Dec	2.1665	.06859	2
	Total	2.4222	.32315	24

Note:

Season abbreviations: aut = autumn, win = winter, spr = spring, sum = summer.

Month abbreviations: Jan = January, Feb = February, Mar = March, Apr = April, May = May, Jun = June, Jul = July, Aug = August, Sep = September, Oct = October, Nov = November, Dec = December.

Indexes: Shannon (*H*) = Shannon–Wiener's diversity index (*H*);

Pielou_e (*J*) = Pielou's evenness index (*J*);

Margalef (*D*) = Margalef's species richness index (*D*).

Table S3. Homogeneity test for Shannon index (*H*).

Test of Homogeneity of Variances					
		Levene Statistic	df1	df2	Sig.
Shannon (*H*)	Based on Mean	.231	11	12	.823

Note:

Indexes: Shannon (*H*) = Shannon–Wiener's diversity index (*H*);

Pielou_e (*J*) = Pielou's evenness index (*J*);

Margalef (*D*) = Margalef's species richness index (*D*).

Table S4. Descriptive statistics of Pielou_e index (*J*).

Descriptive Statistics				
Dependent Variable: Pielou_e (*J*)				
Season	Month	Mean	Std. Deviation	N
aut	Sep	.7150	.01251	2
	Oct	.7342	.03514	2
	Nov	.6432	.03158	2
	Total	.6975	.04818	6
win	Jan	.5966	.03645	2
	Feb	.6101	.02359	2
	Dec	.7055	.06314	2
	Total	.6374	.06320	6
spr	Mar	.7883	.05171	2
	Apr	.7507	.01814	2
	May	.7726	.02688	2
	Total	.7706	.03210	6
sum	Jun	.7247	.00893	2
	Jul	.7333	.02048	2
	Aug	.7366	.01842	2
	Total	.7315	.01406	6
Total	Jan	.5966	.03645	2
	Feb	.6101	.02359	2
	Mar	.7883	.05171	2
	Apr	.7507	.01814	2
	May	.7726	.02688	2
	Jun	.7247	.00893	2
	Jul	.7333	.02048	2
	Aug	.7366	.01842	2
	Sep	.7150	.01251	2
	Oct	.7342	.03514	2
	Nov	.6432	.03158	2
	Dec	.7055	.06314	2
	Total	.7092	.06429	24

Note:

Season abbreviations: aut = autumn, win = winter, spr = spring, sum = summer.

Month abbreviations: Jan = January, Feb = February, Mar = March, Apr = April, May = May, Jun = June, Jul = July, Aug = August, Sep = September, Oct = October, Nov = November, Dec = December.

Indexes: Shannon (*H*) = Shannon–Wiener's diversity index (*H*);

Pielou_e (*J*) = Pielou's evenness index (*J*);

Margalef (*D*) = Margalef's species richness index (*D*).

Table S5. Homogeneity test for Pielou_e index (*J*).

Test of Homogeneity of Variances					
		Levene Statistic	df1	df2	Sig.
Pielou_e (*J*)	Based on Mean	.627	11	12	.554

Note:

Indexes: Shannon (*H*) = Shannon–Wiener's diversity index (*H*);

Pielou_e (*J*) = Pielou's evenness index (*J*);

Margalef (*D*) = Margalef's species richness index (*D*).

Table S6. Descriptive statistics of Margalef index (*D*).

Descriptive Statistics				
Dependent Variable: Margalef (*D*)				
Season	Month	Mean	Std. Deviation	N
aut	Sep	4.0964	.27828	2
	Oct	3.1463	.57338	2
	Nov	3.2928	.06813	2
	Total	3.5118	.53988	6
win	Jan	2.2261	.20277	2
	Feb	2.5142	.42673	2
	Dec	3.5960	.90646	2
	Total	2.7788	.79141	6
spr	Mar	2.0970	.07928	2
	Apr	2.9033	.39459	2
	May	3.8370	.68885	2
	Total	2.9457	.85670	6
sum	Jun	4.2944	.19532	2
	Jul	3.4002	.57072	2
	Aug	4.4573	.26435	2
	Total	4.0506	.58815	6
Total	Jan	2.2261	.20277	2
	Feb	2.5142	.42673	2
	Mar	2.0970	.07928	2
	Apr	2.9033	.39459	2
	May	3.8370	.68885	2
	Jun	4.2944	.19532	2
	Jul	3.4002	.57072	2
	Aug	4.4573	.26435	2
	Sep	4.0964	.27828	2
	Oct	3.1463	.57338	2
	Nov	3.2928	.06813	2
	Dec	3.5960	.90646	2
	Total	3.3217	.83432	24

Note:

Season abbreviations: aut = autumn, win = winter, spr = spring, sum = summer.

Month abbreviations: Jan = January, Feb = February, Mar = March, Apr = April, May = May, Jun = June, Jul = July, Aug = August, Sep = September, Oct = October, Nov = November, Dec = December.

Indexes: Shannon (*H*) = Shannon–Wiener's diversity index (*H*);

Pielou_e (*J*) = Pielou's evenness index (*J*); Margalef (*D*) = Margalef's species richness index (*D*).

Table S7. Homogeneity test for Margalef index (*D*).

Test of Homogeneity of Variances					
		Levene Statistic	df1	df2	Sig.
Margalef (*D*)	Based on Mean	.713	11	12	.424

Note:

Indexes: Shannon (*H*) = Shannon–Wiener's diversity index (*H*);

Pielou_e (*J*) = Pielou's evenness index (*J*);

Margalef (*D*) = Margalef's species richness index (*D*).

Table S8. Normality test for environmental and nekton variables in autumn and spring.

Tests of Normality							
	VAR00001	Kolmogorov-Smirnov[a]			Shapiro-Wilk		
		Statistical data	df	significance	Statistical data	df	significance
temp	aut	.213	12	.138	.744	12	.002
	spr	.234	12	.067	.869	12	.063
sal	aut	.221	12	.121	.725	12	.001
	spr	.227	12	.088	.873	12	.071
pH	aut	.197	12	.200*	.880	12	.087
	spr	.157	12	.200*	.931	12	.395
DO	aut	.214	12	.139	.756	12	.003
	spr	.212	12	.136	.803	12	.010
COD	aut	.177	12	.200*	.907	12	.198
	spr	.236	12	.070	.650	12	.000
DIP	aut	.189	12	.200*	.923	12	.314
	spr	.195	12	.200*	.946	12	.577
DIN	aut	.213	12	.138	.894	12	.131
	spr	.134	12	.200*	.950	12	.633
Si	aut	.254	12	.031	.895	12	.138
	spr	.234	12	.068	.867	12	.059
DEEP	aut	.162	12	.200*	.898	12	.150
	spr	.204	12	.180	.879	12	.086
biomass	aut	.220	12	.116	.600	12	.000
	spr	.219	12	.117	.929	12	.365
abundance	aut	.212	12	.136	.590	12	.000
	spr	.210	12	.149	.927	12	.348
C	aut	.198	12	.200*	.936	12	.450
	spr	.237	12	.060	.810	12	.012
Shannon (*H*)	aut	.117	12	.200*	.928	12	.357
	spr	.200	12	.198	.837	12	.026
Pielou_e (*J*)	aut	.192	12	.200*	.906	12	.192
	spr	.126	12	.200*	.936	12	.447
Margalef (*D*)	aut	.148	12	.200*	.935	12	.433
	spr	.261	12	.183	.820	12	.182
*. This is the significant lower bound of true.							
[a]. Lilliefors Significance Correction							

Note:

Season abbreviations: aut = autumn, win = winter, spr = spring, sum = summer.

Indexes: Shannon (*H*) = Shannon–Wiener's diversity index (*H*);

Pielou_e (*J*) = Pielou's evenness index (*J*);

Margalef (*D*) = Margalef's species richness index (*D*).

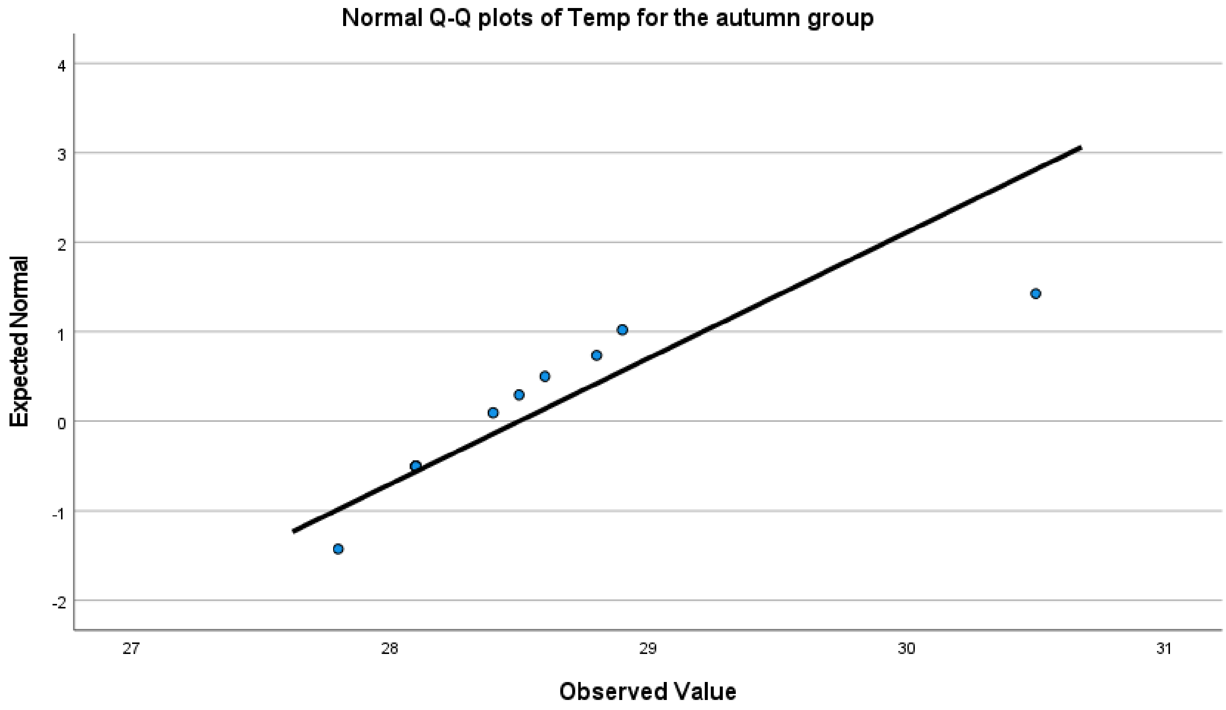

Figure S13. Normal Q-Q plots of temp for the autumn group.

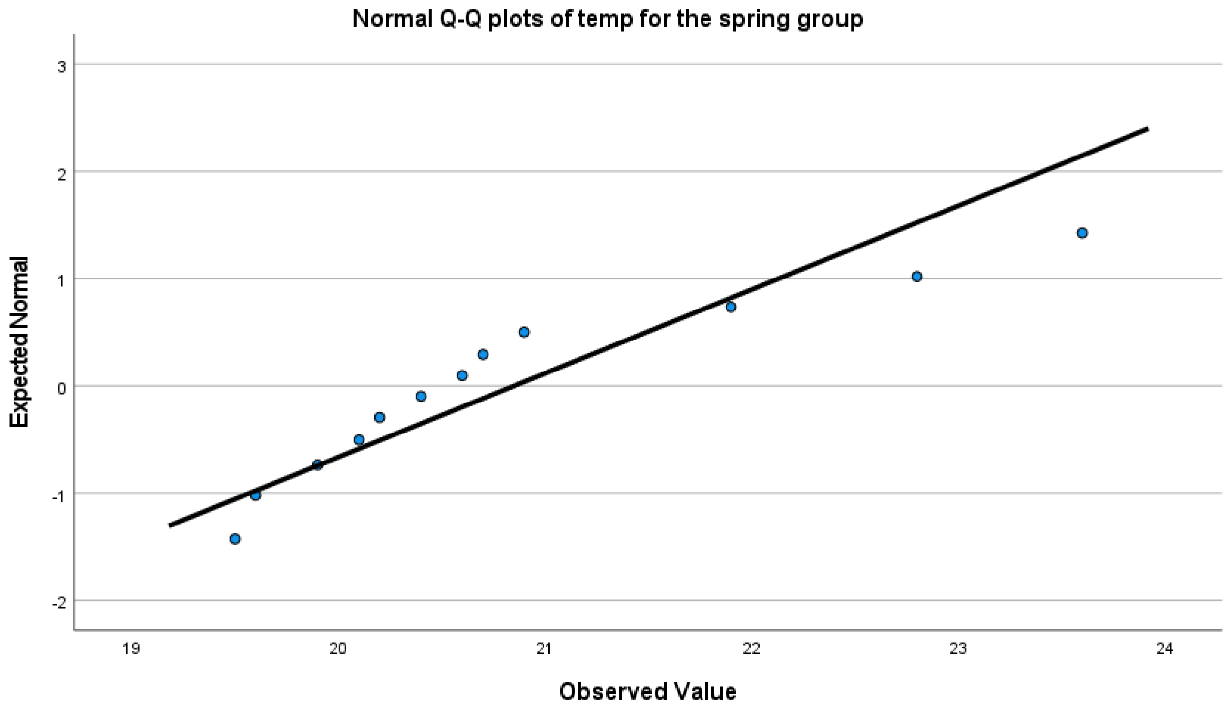

Figure S14. Normal Q-Q plots of temp for the spring group.

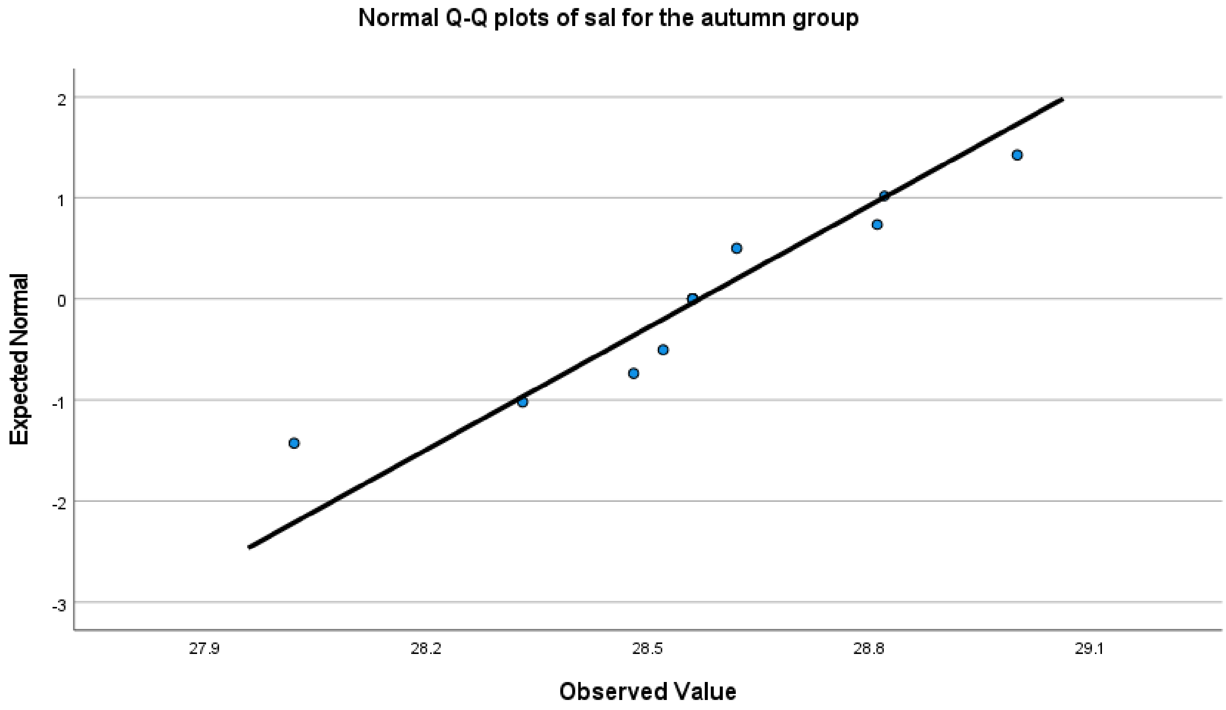

Figure S15. Normal Q-Q plots of sal for the autumn group.

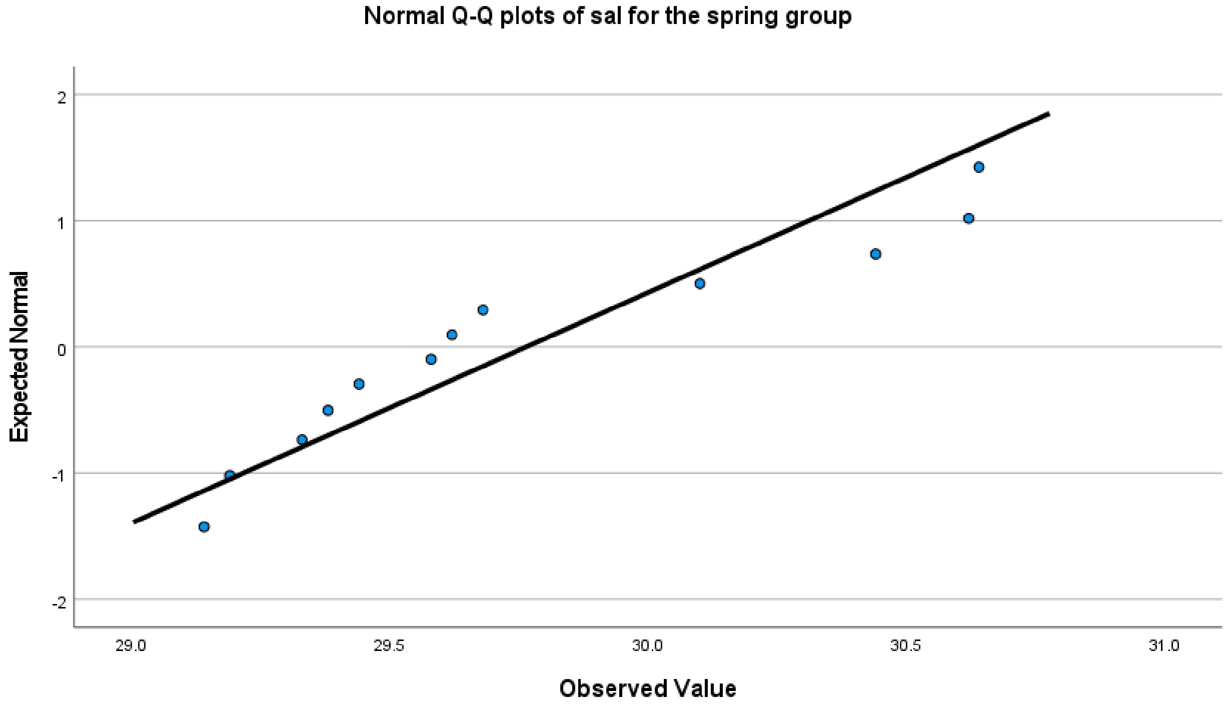

Figure S16. Normal Q-Q plots of sal for the spring group.

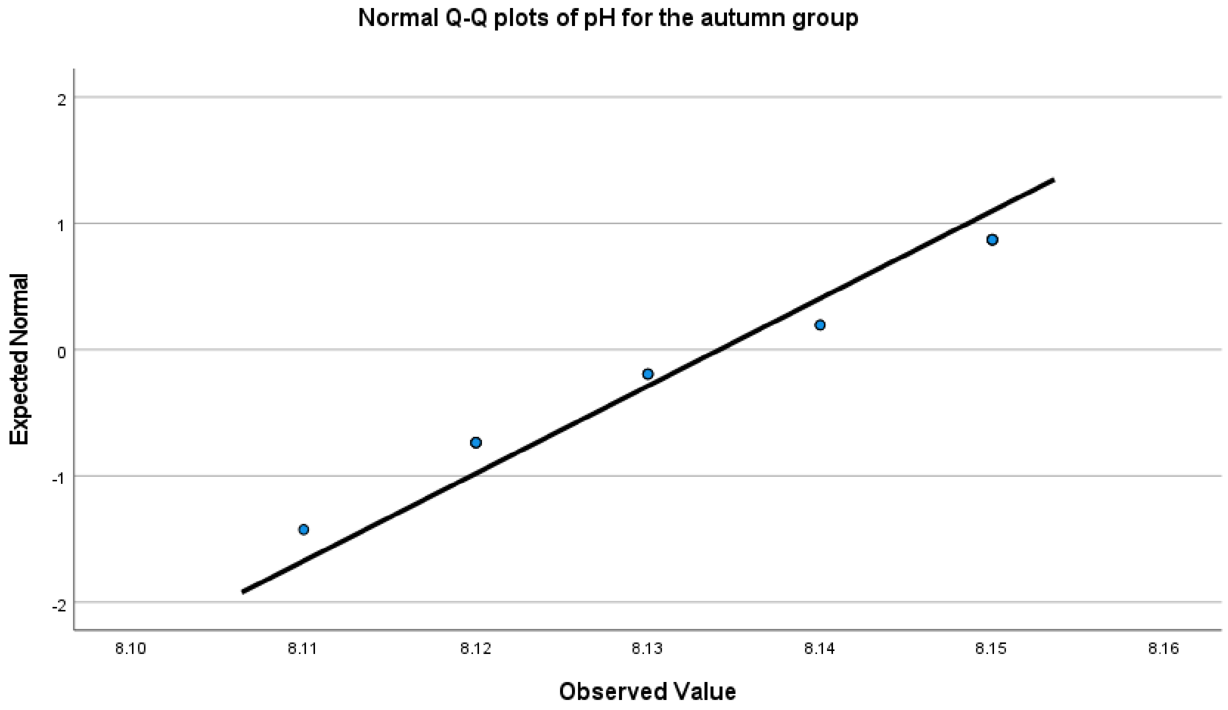

Figure S17. Normal Q-Q plots of pH for the autumn group.

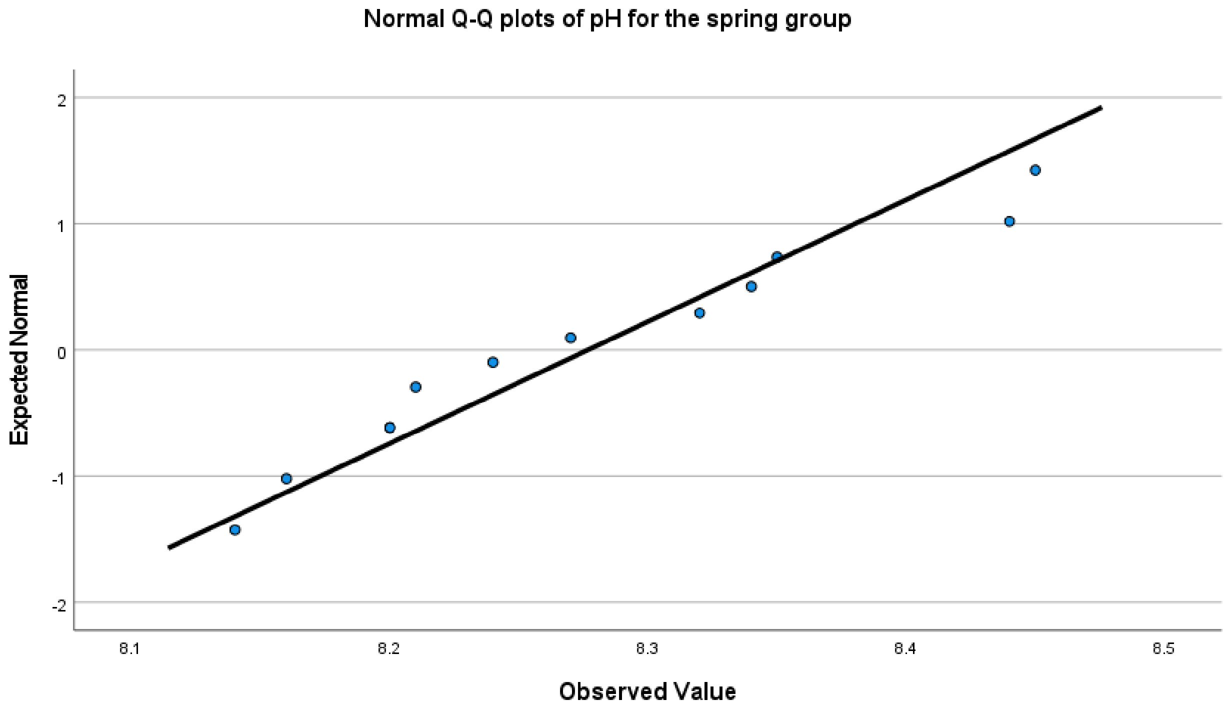

Figure S18. Normal Q-Q plots of pH for the spring group.

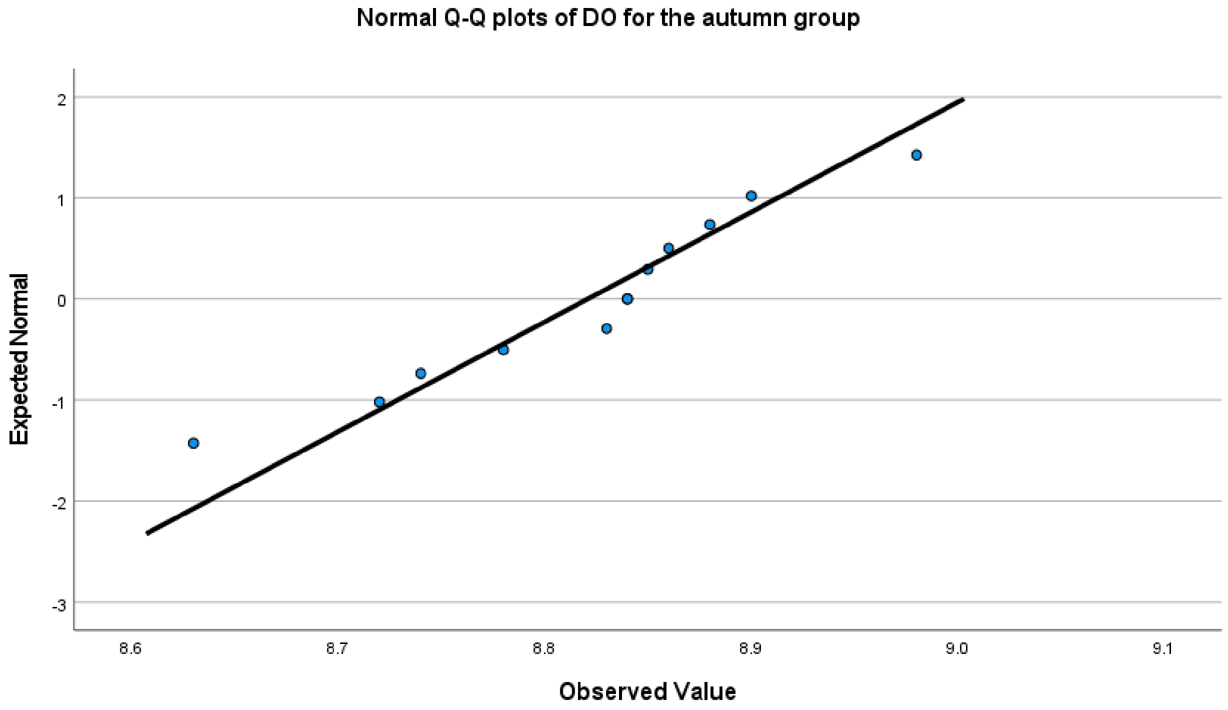

Figure S19. Normal Q-Q plots of DO for the autumn group.

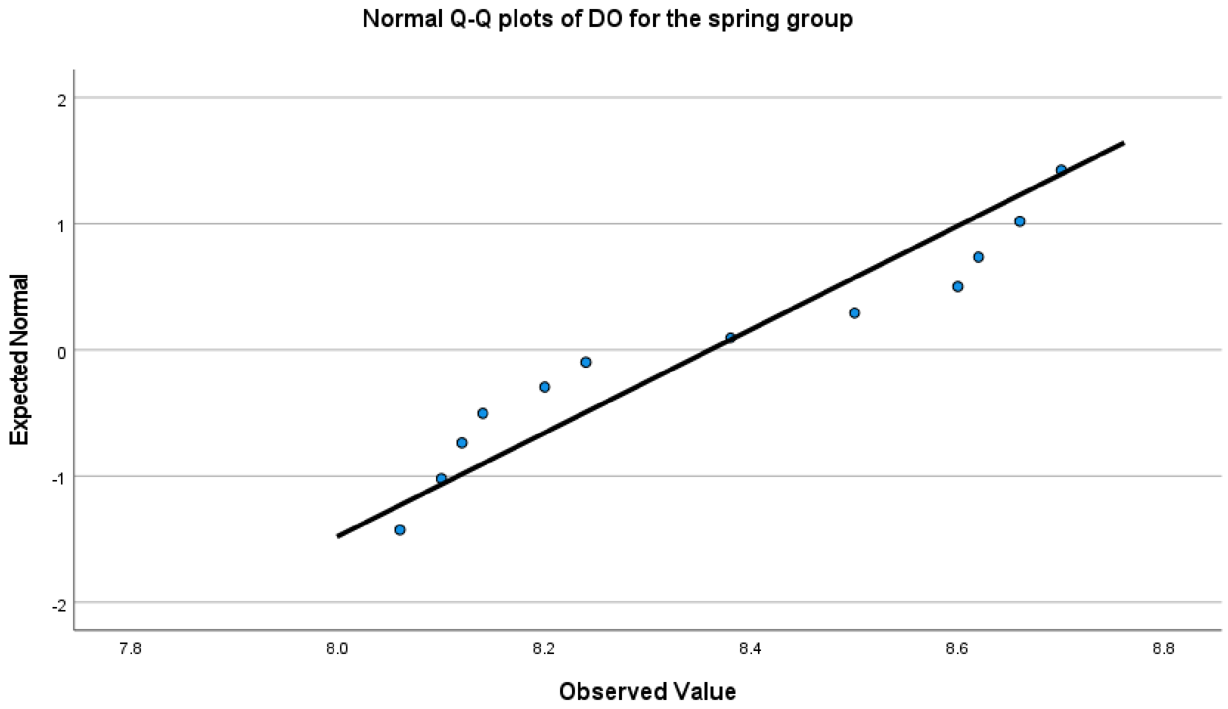

Figure S20. Normal Q-Q plots of DO for the spring group.

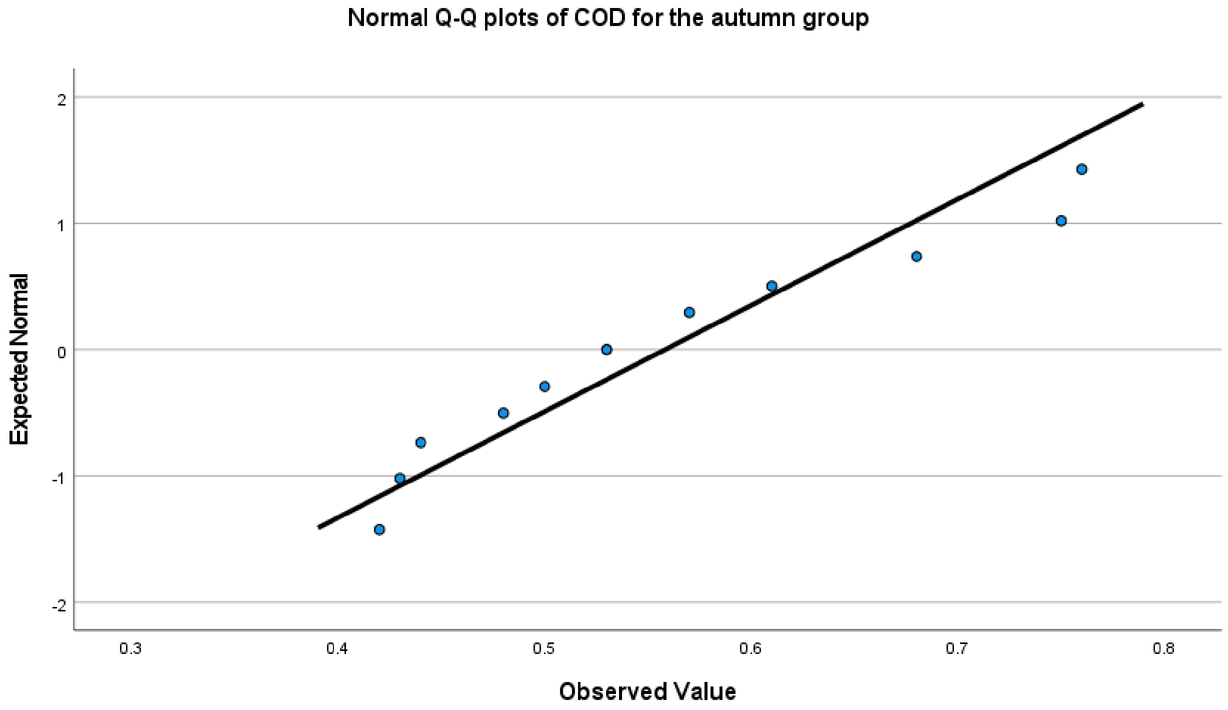

Figure S21. Normal Q-Q plots of COD for the autumn group.

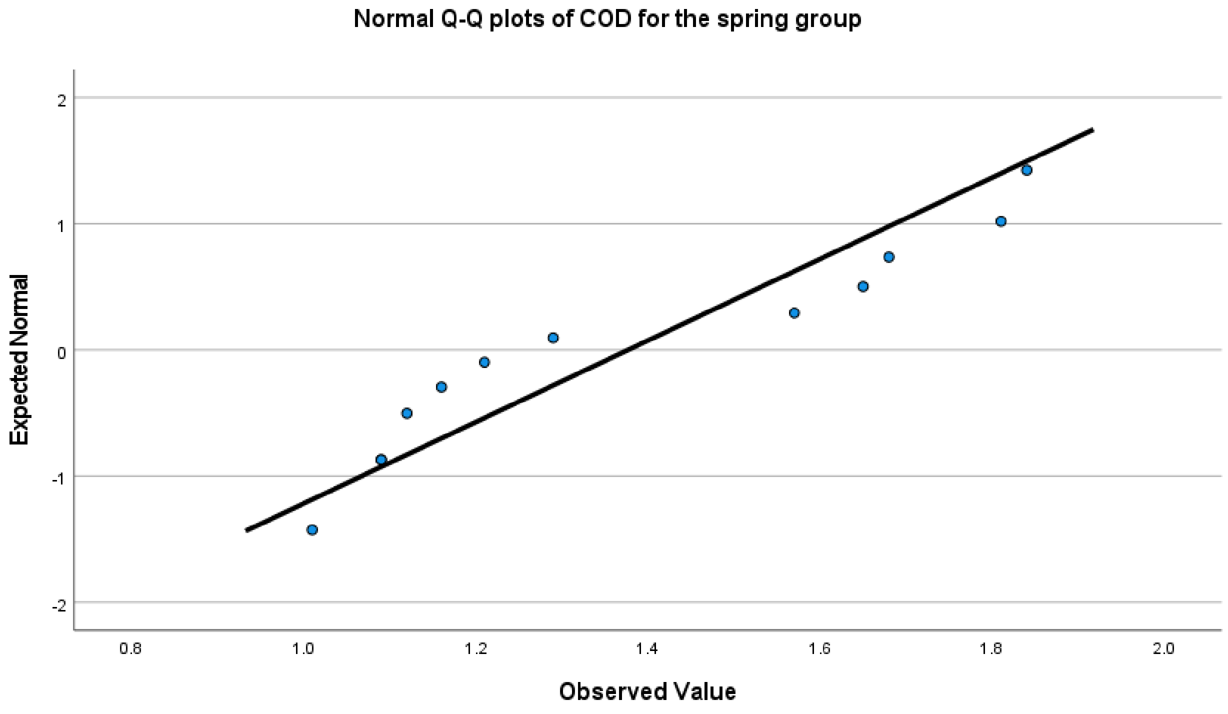

Figure S22. Normal Q-Q plots of COD for the spring group.

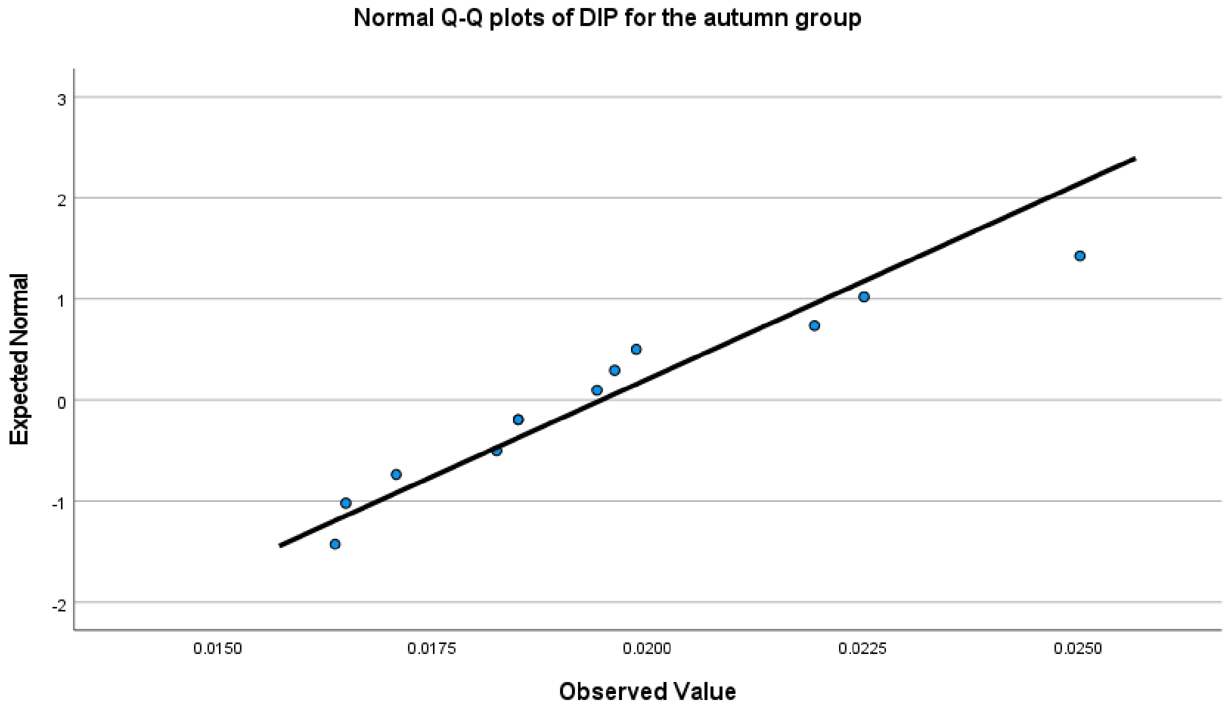

Figure S23. Normal Q-Q plots of DIP for the autumn group.

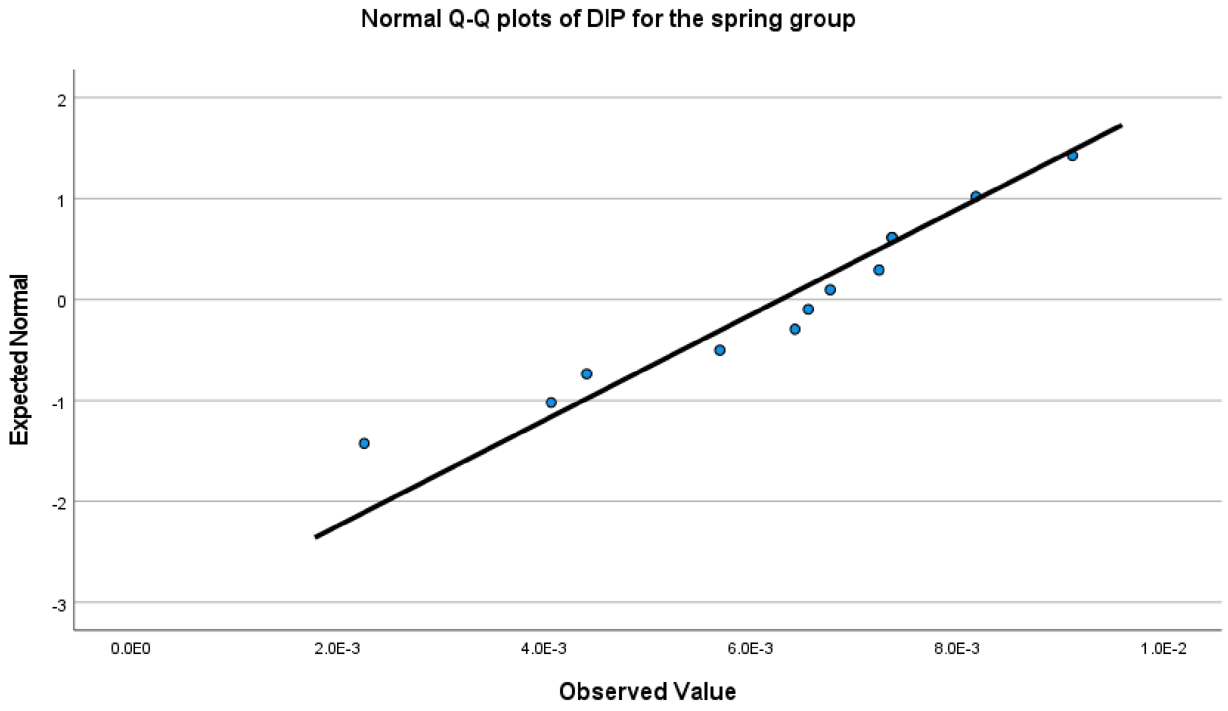

Figure S24. Normal Q-Q plots of DIP for the spring group.

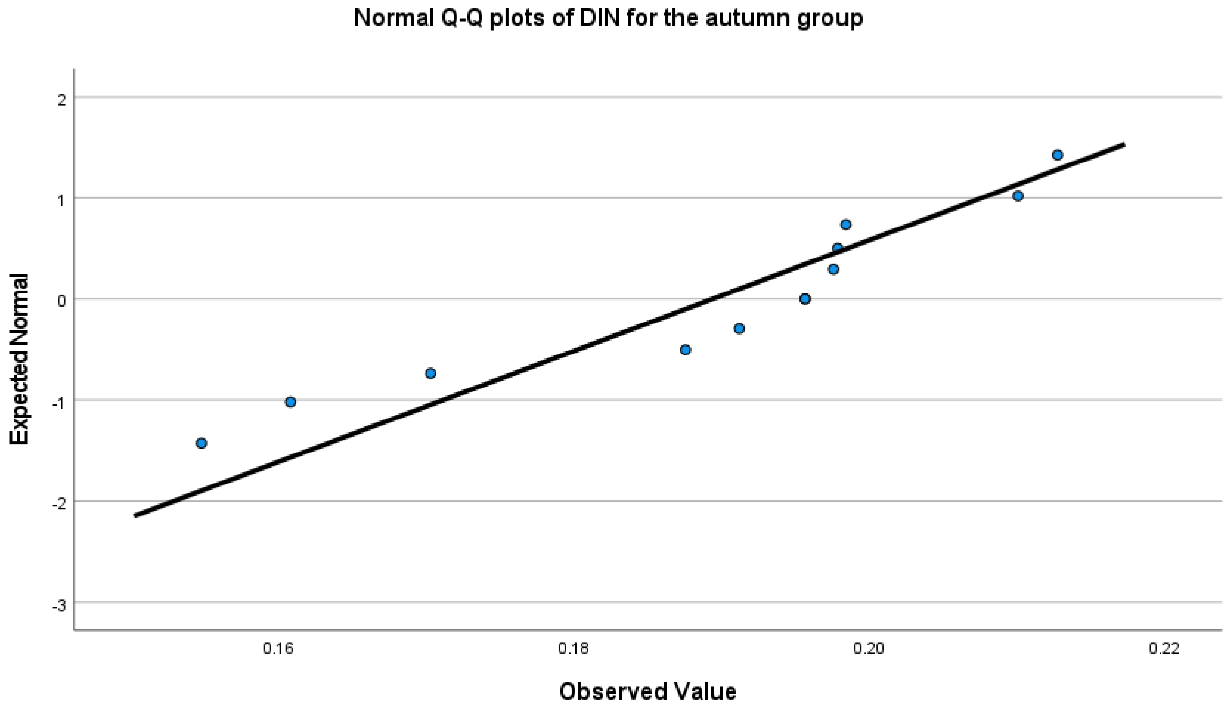

Figure S25. Normal Q-Q plots of DIN for the autumn group.

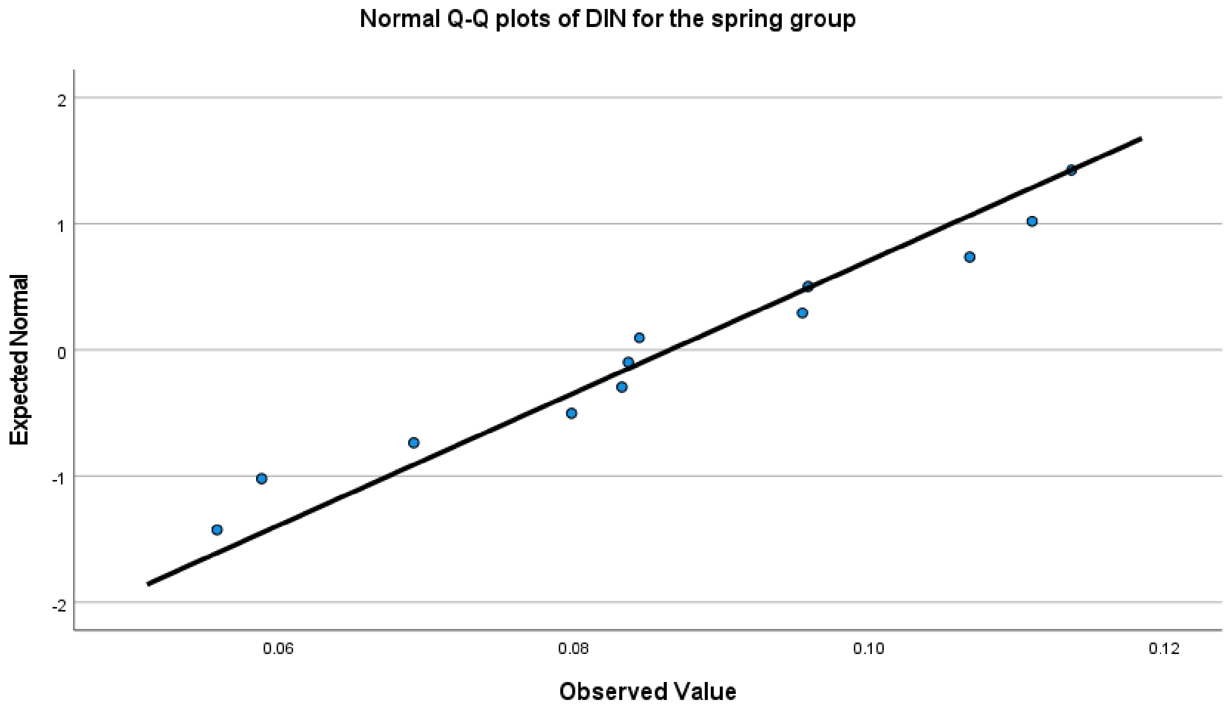

Figure S26. Normal Q-Q plots of DIN for the spring group.

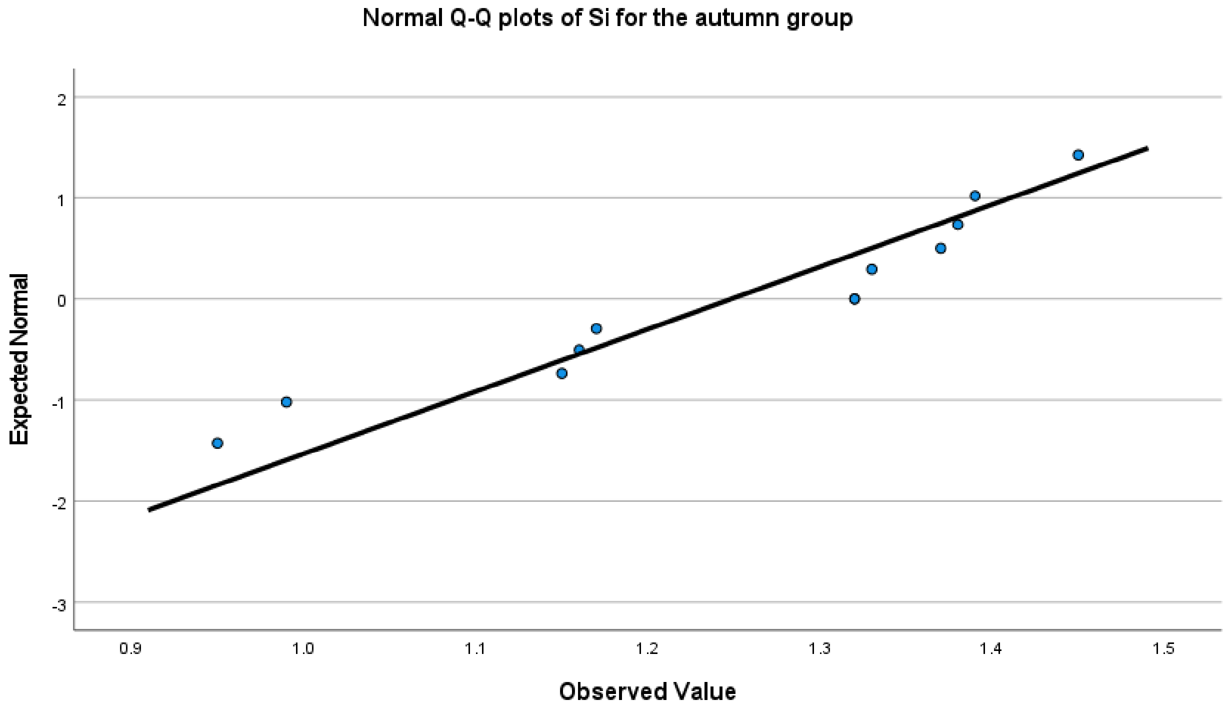

Figure S27. Normal Q-Q plots of Si for the autumn group.

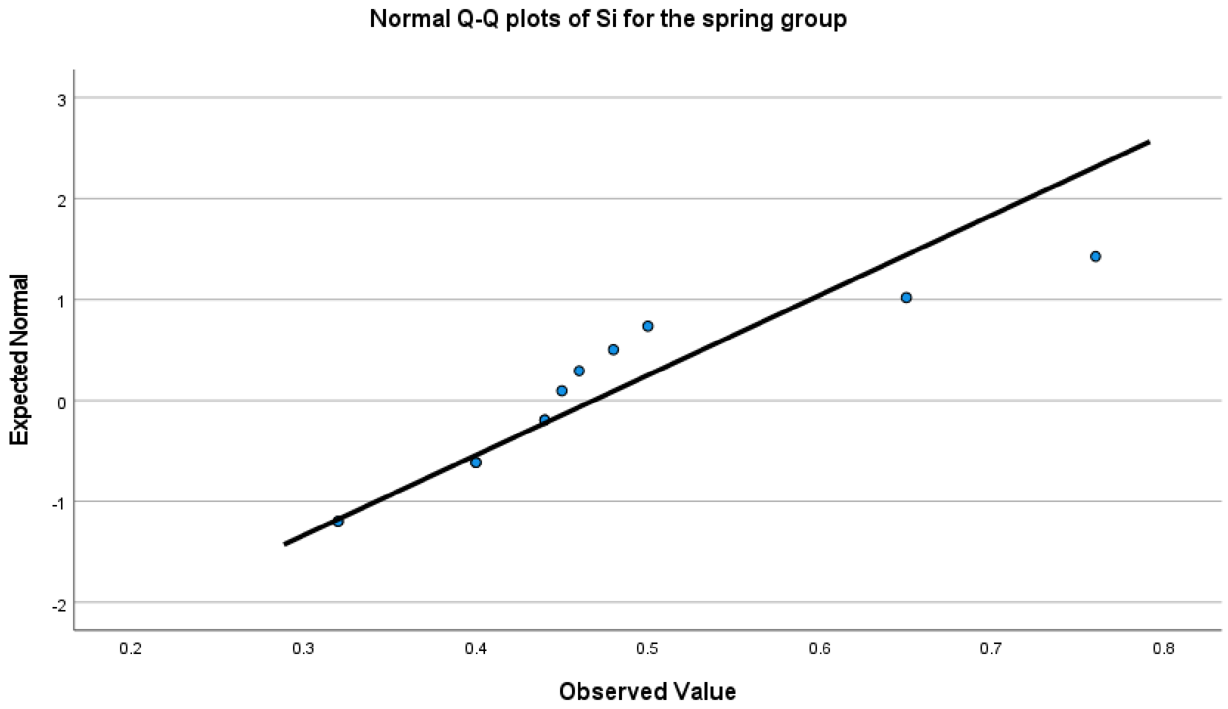

Figure S28. Normal Q-Q plots of Si for the spring group.

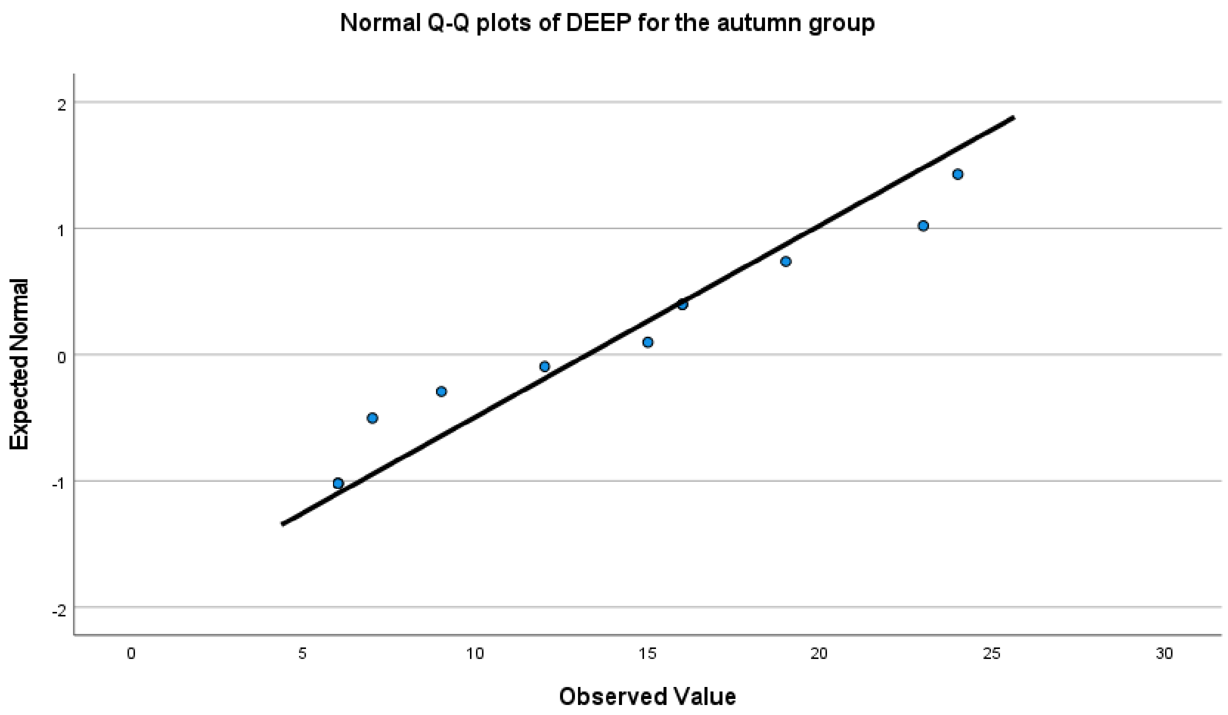

Figure S29. Normal Q-Q plots of DEEP for the autumn group.

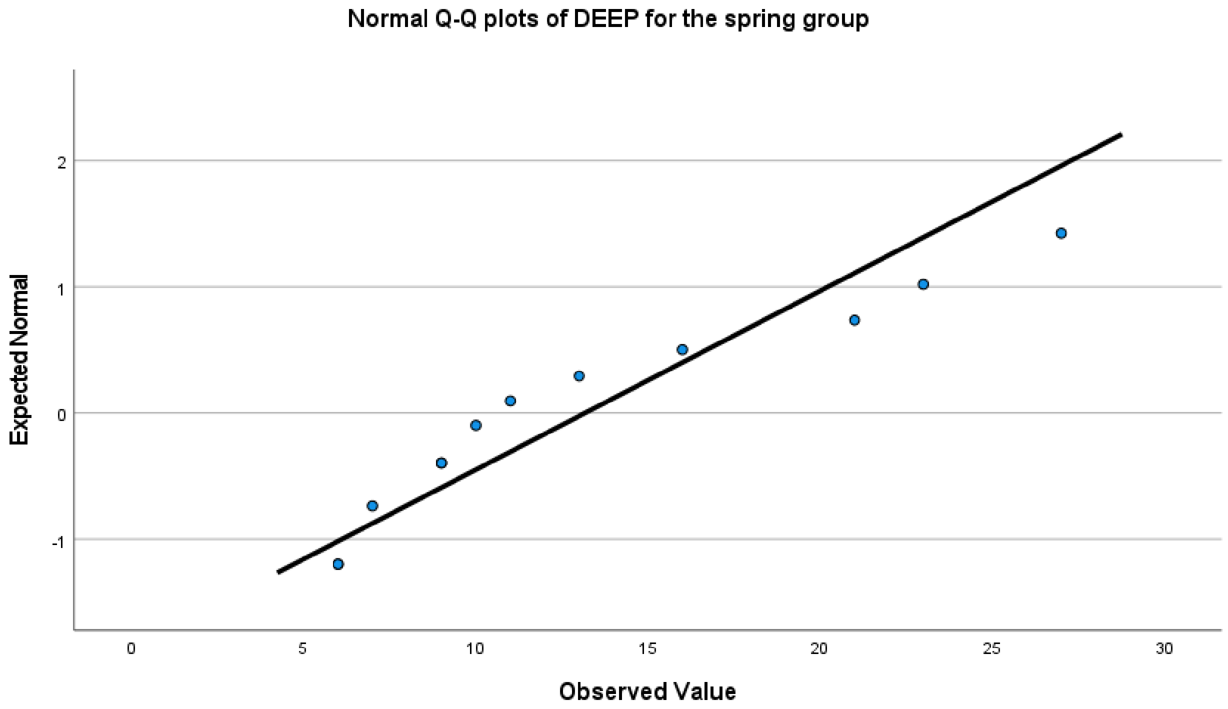

Figure S30. Normal Q-Q plots of DEEP for the spring group.

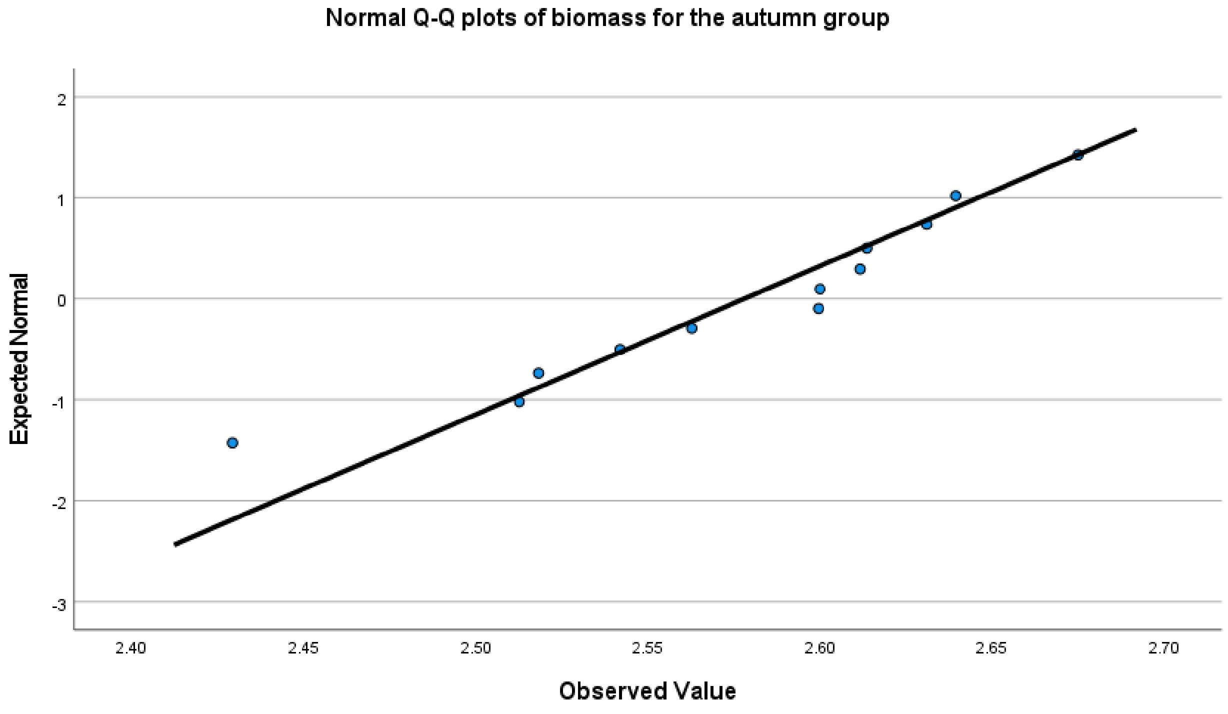

Figure S31. Normal Q-Q plots of biomass for the autumn group.

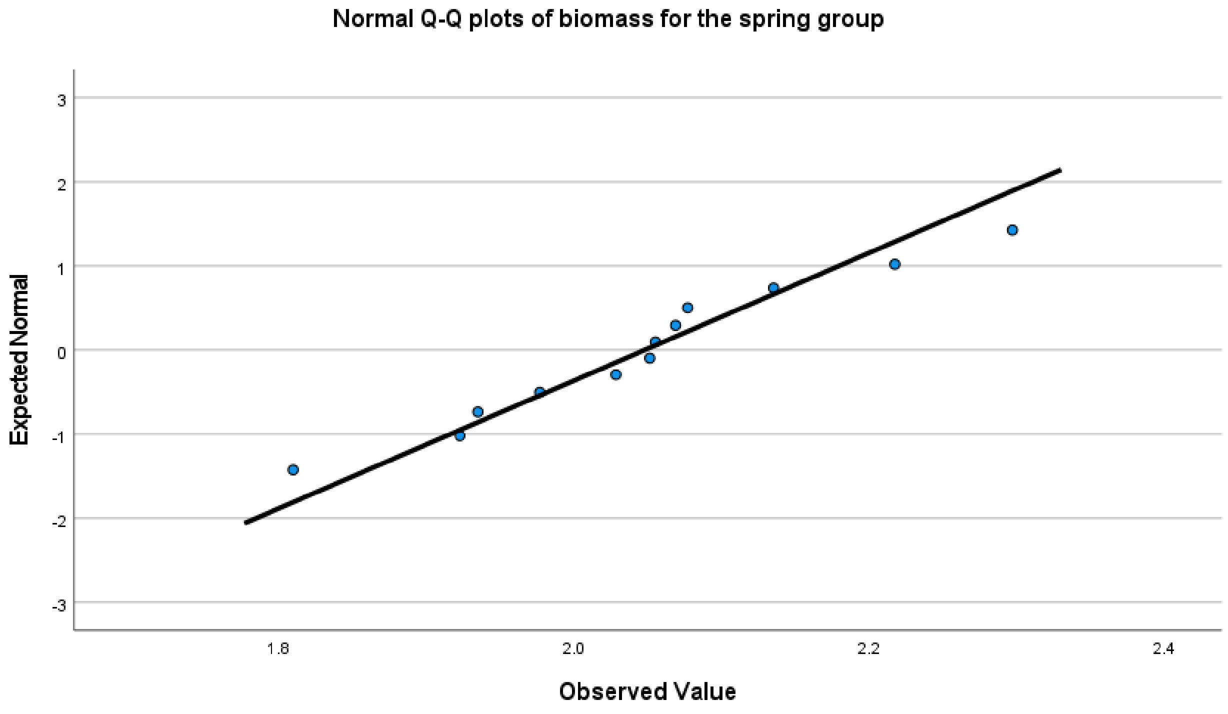

Figure S32. Normal Q-Q plots of biomass for the spring group.

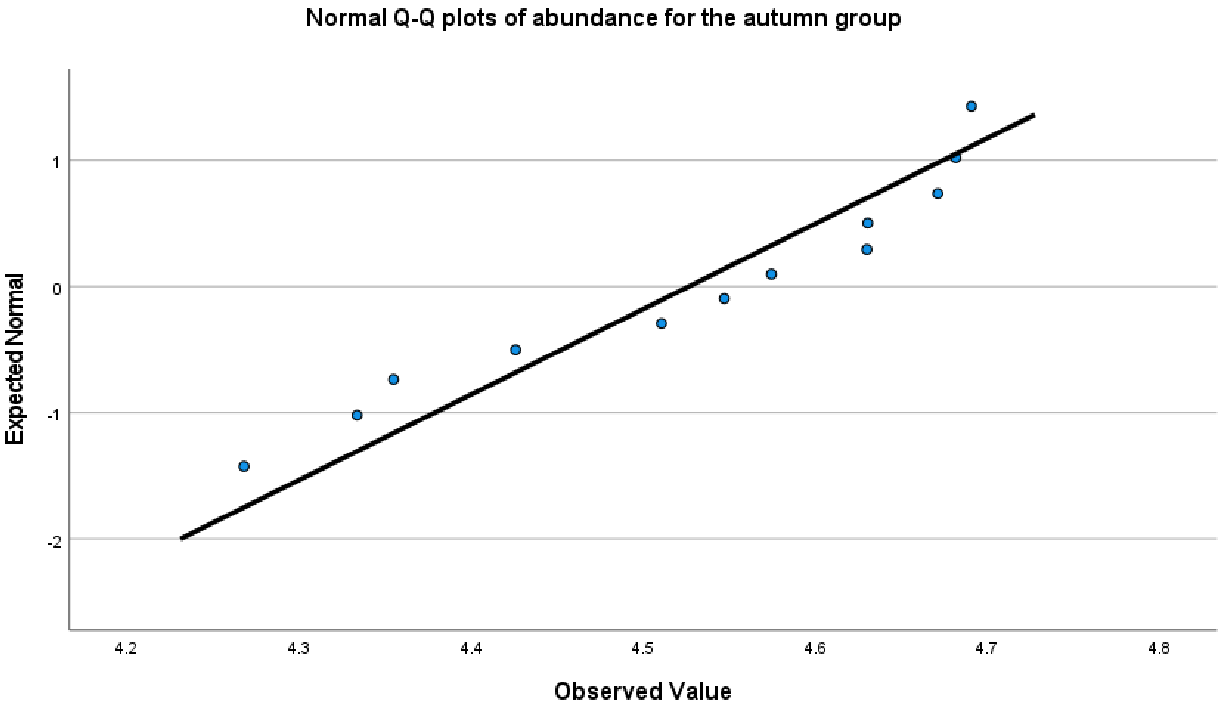

Figure S33. Normal Q-Q plots of abundance for the autumn group.

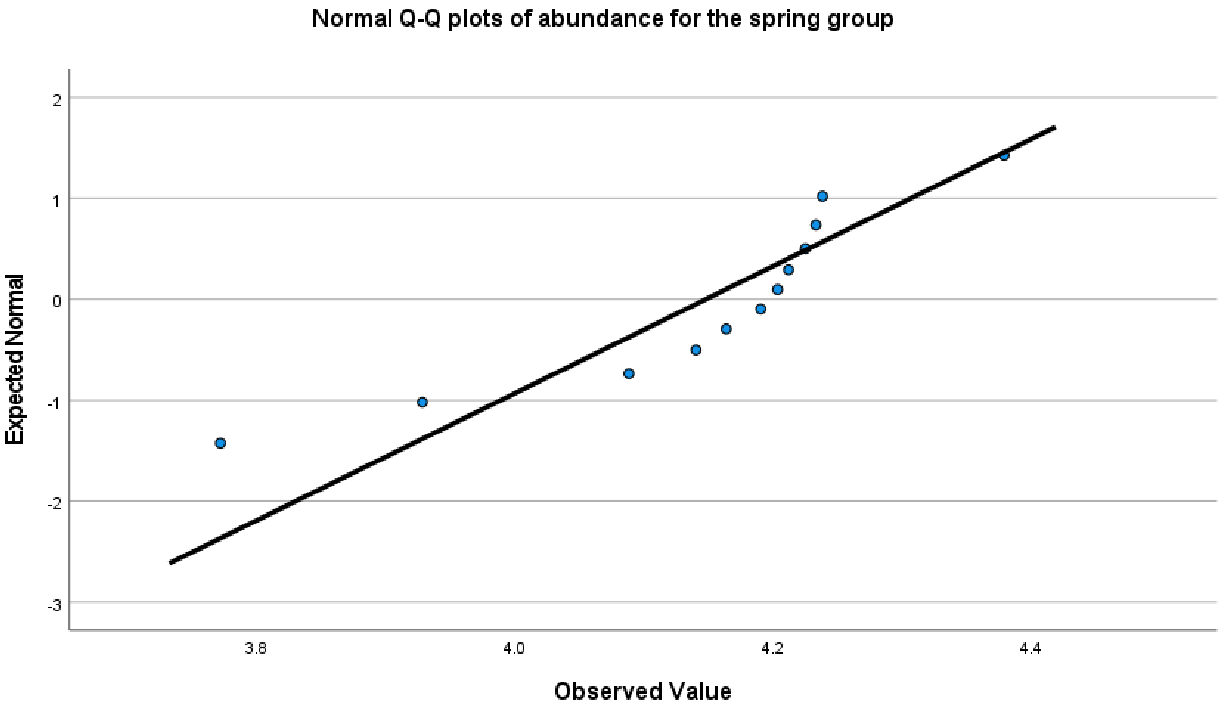

Figure S34. Normal Q-Q plots of abundance for the spring group.

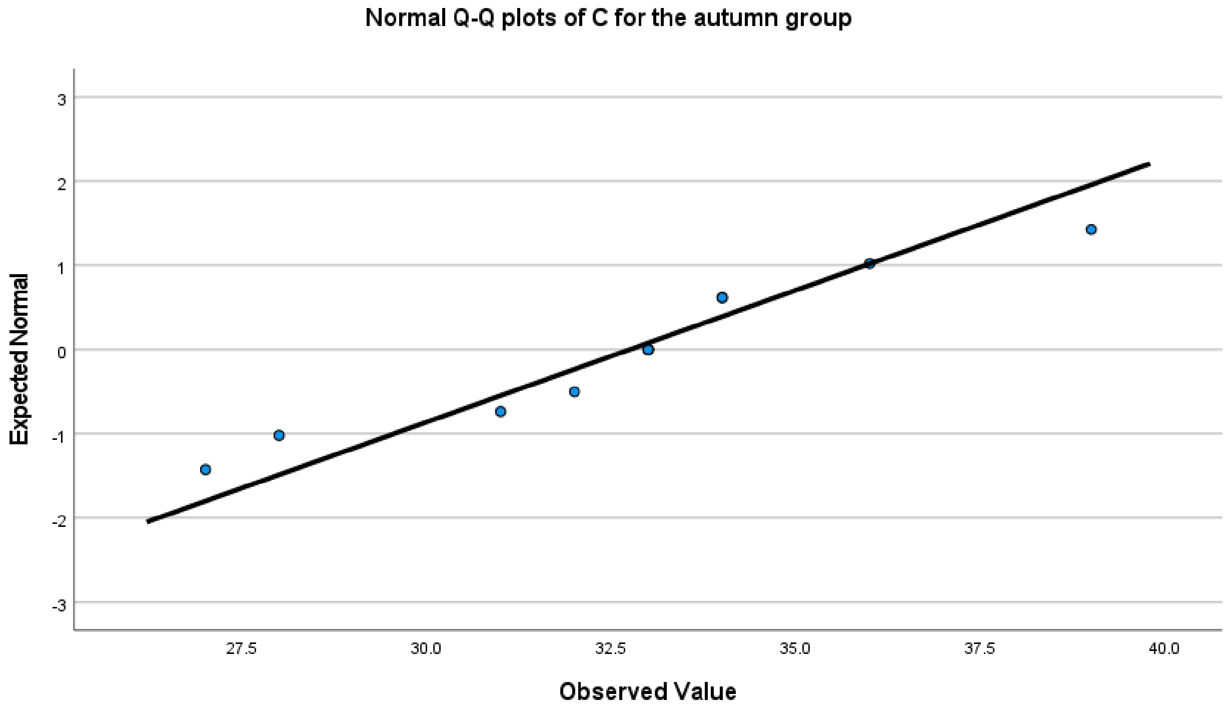

Figure S35. Normal Q-Q plots of *C* for the autumn group.

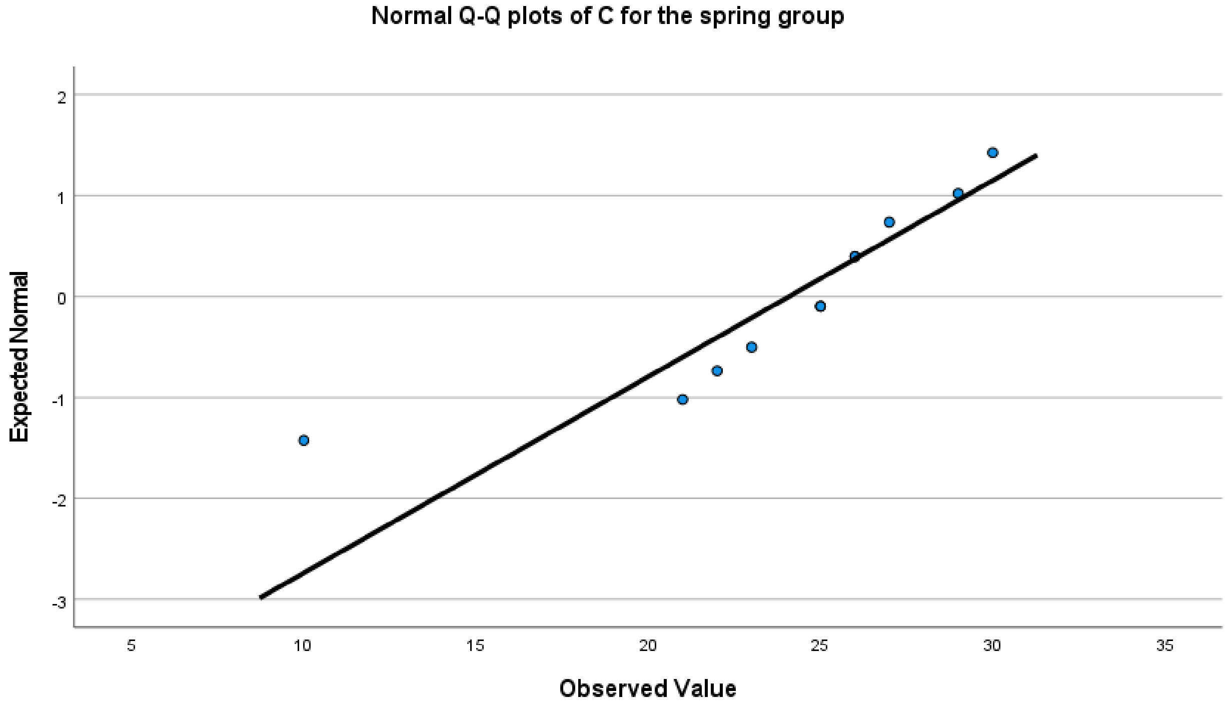

Figure S36. Normal Q-Q plots of *C* for the spring group.

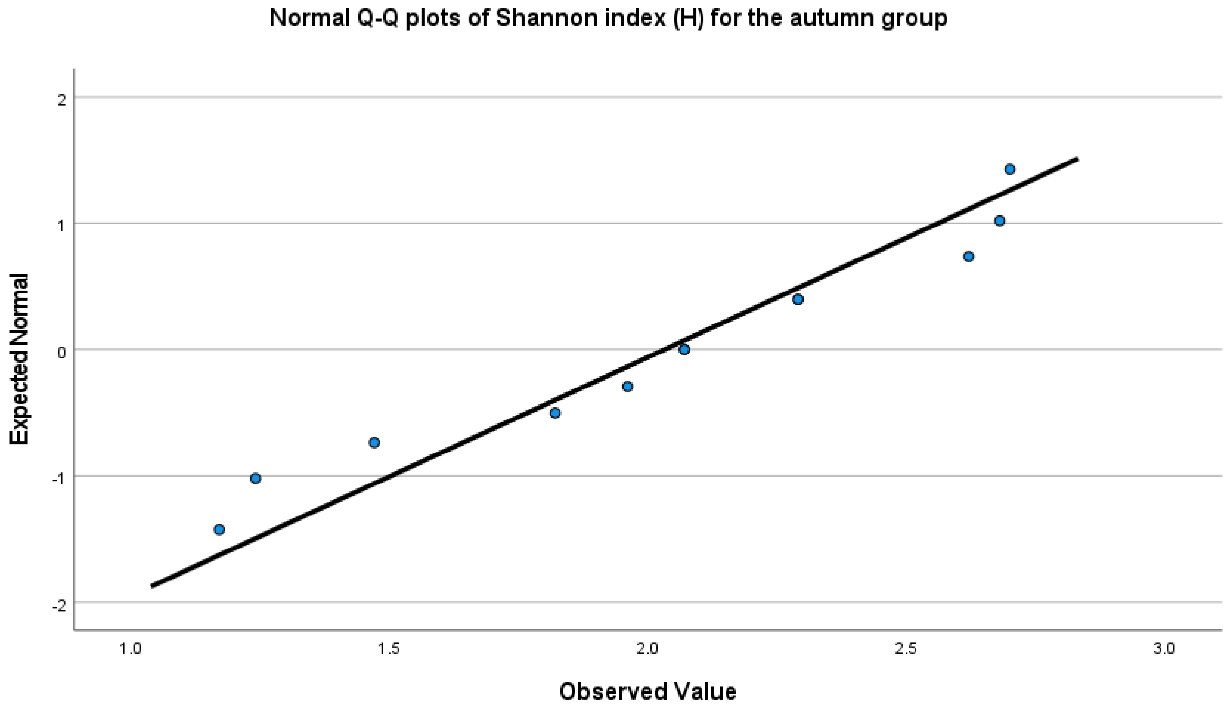

Figure S37. Normal Q-Q plots of Shannon index (*H*) for the autumn group.

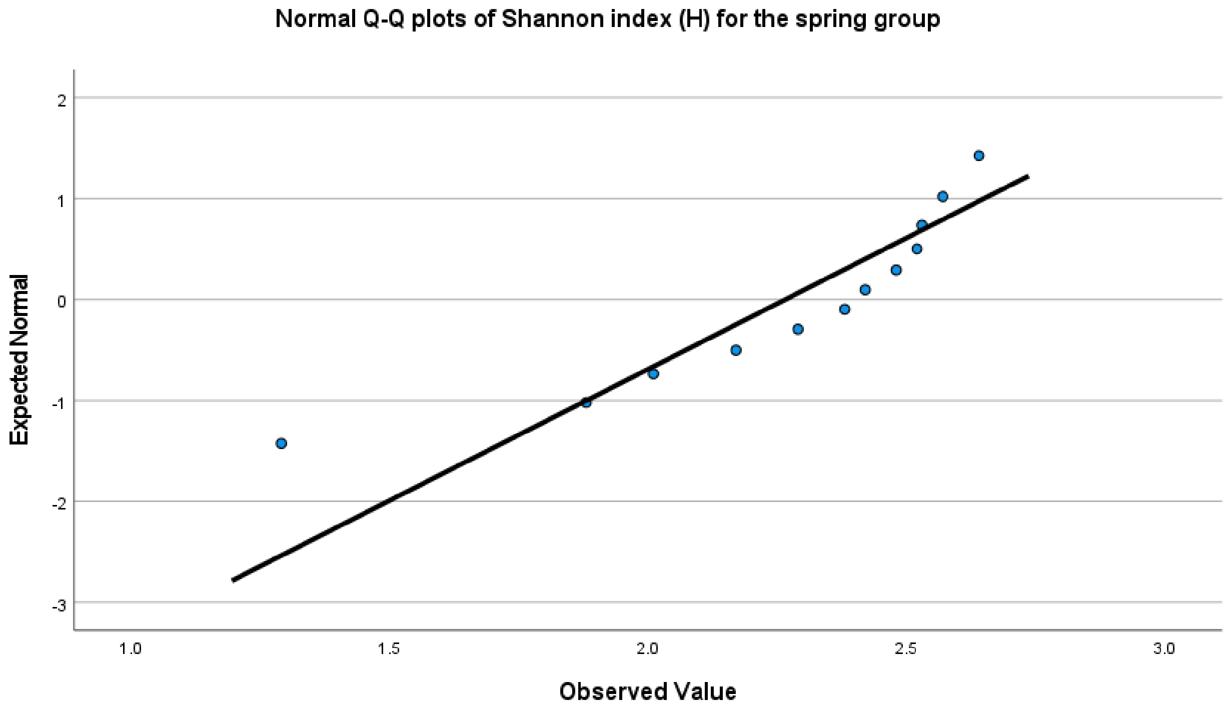

Figure S38. Normal Q-Q plots of Shannon index (*H*) for the spring group.

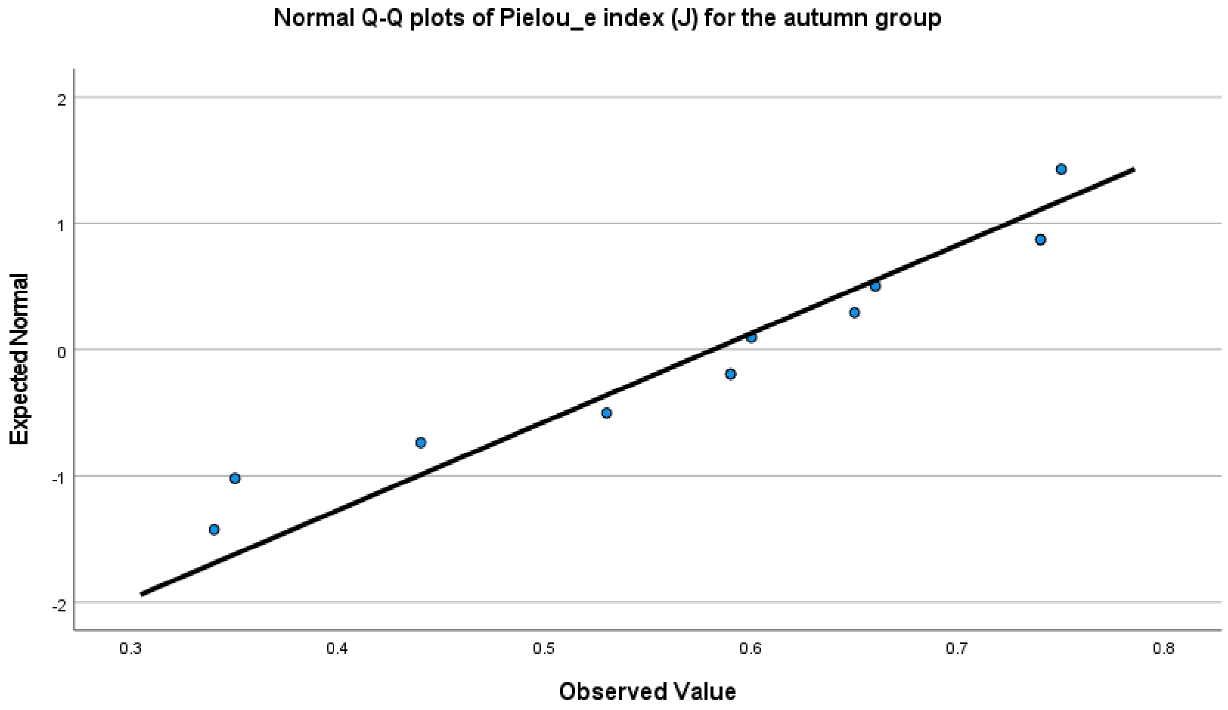

Figure S39. Normal Q-Q plots of Pielou_e index (*J*) for the autumn group.

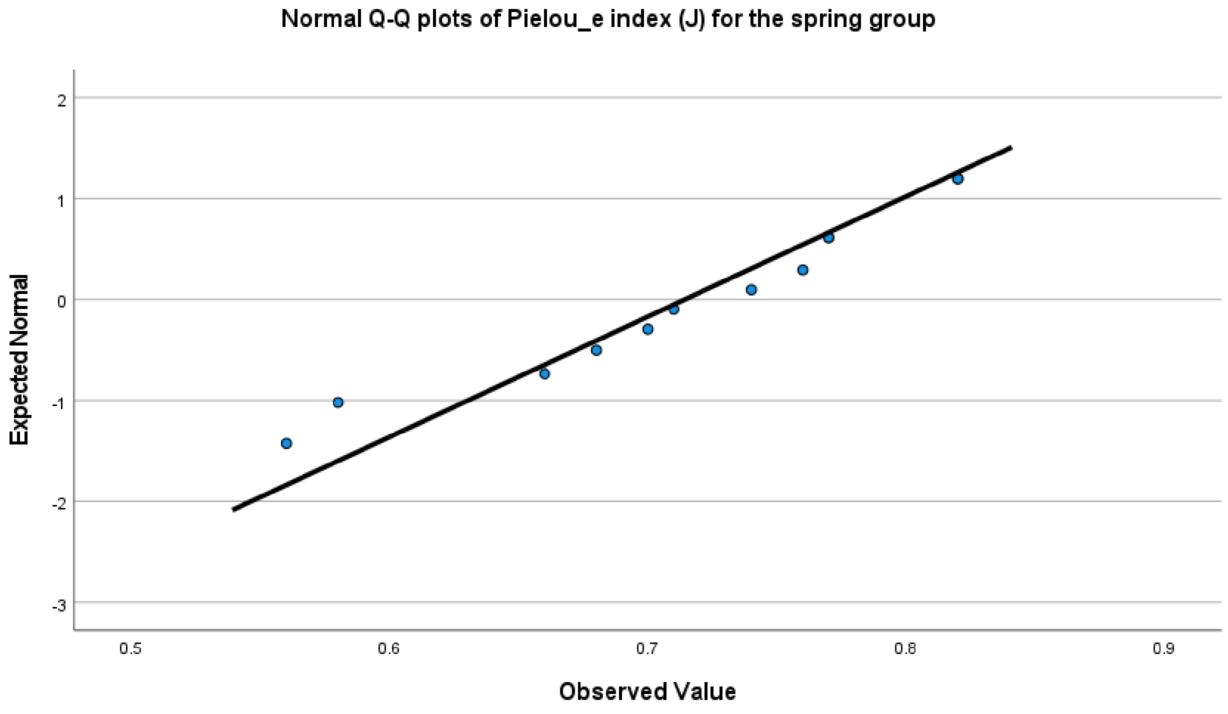

Figure S40. Normal Q-Q plots of Pielou_e index (*J*) for the spring group.

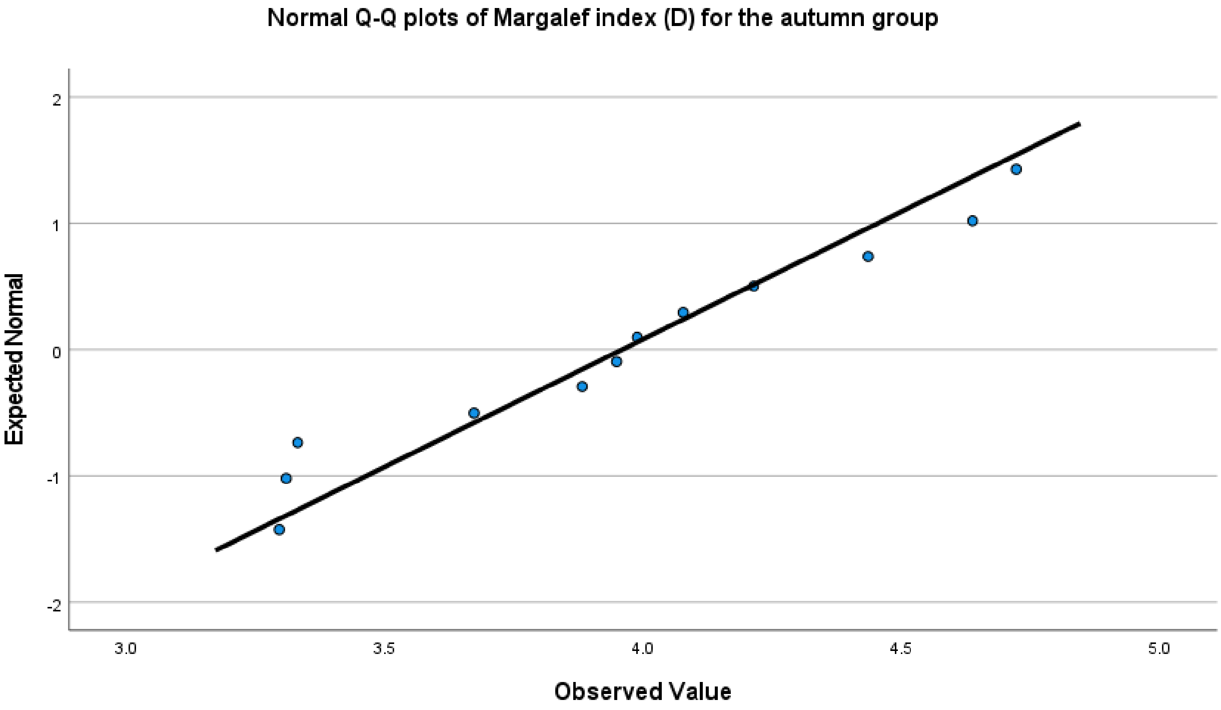

Figure S41. Normal Q-Q plots of Margalef index (*D*) for the autumn group.

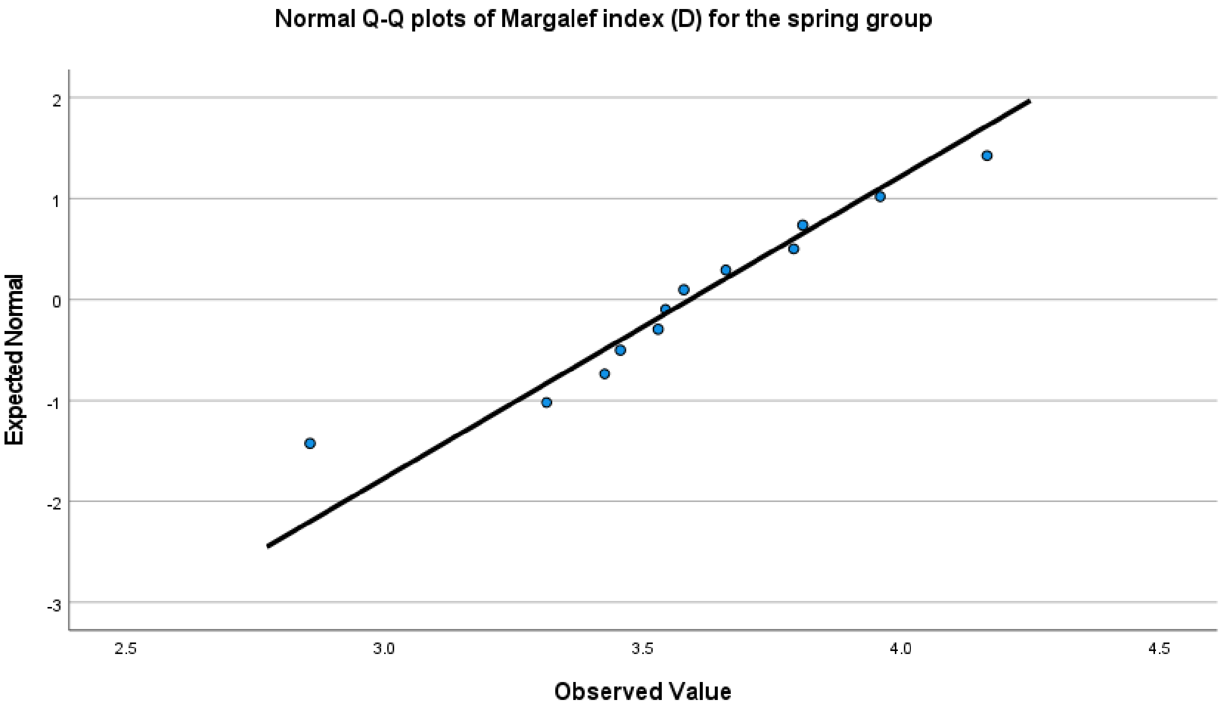

Figure S42. Normal Q-Q plots of Margalef index (*D*) for the spring group.

Table S9. Homogeneity test for environmental and nekton variables in autumn and spring.

Test of Homogeneity of Variances				
	Levene	df1	df2	significance
temp	2.887	1	22	.103
sal	.027	1	22	.871
pH	3.301	1	22	.072
DO	1.414	1	22	.211
COD	1.215	1	22	.233
DIP	.722	1	22	.405
DIN	.057	1	22	.814
Si	.076	1	22	.785
DEEP	.112	1	22	.741
biomass	2.292	1	22	.144
abundance	2.546	1	22	.125
C	2.309	1	22	.143
Shannon (*H*)	1.600	1	22	.219
Pielou_e (*J*)	3.763	1	22	.065
Margalef (*D*)	.263	1	22	.613

Note:

Season abbreviations: aut = autumn, win = winter, spr = spring, sum = summer.

Indexes: Shannon (*H*) = Shannon–Wiener's diversity index (*H*);

Pielou_e (*J*) = Pielou's evenness index (*J*);

Margalef (*D*) = Margalef's species richness index (*D*).

Table S10. Nested ANOVA of Shannon index (*H*).

Tests of Between-Subjects Effects					
Dependent Variable: Shannon (*H*)					
Source	Type III Sum of Squares	df	Mean Square	F	Sig.
Corrected Model	2.145[a]	11	.195	9.116	.000
Intercept	140.810	1	140.810	6582.519	.000
Month (Season)	.443	8	.055	2.591	.067
Season	1.702	3	.567	26.516	.000
Error	.257	12	.021		
Total	143.212	24			
Corrected Total	2.402	23			
[a]. R Squared = .893 (Adjusted R Squared = .795)					

Note:

Indexes: Shannon (*H*) = Shannon–Wiener's diversity index (*H*);

Pielou_e (*J*) = Pielou's evenness index (*J*);

Margalef (*D*) = Margalef's species richness index (*D*).

Table S11. Multiple comparisons for Shannon index (*H*).

Multiple Comparisons						
Dependent Variable: Shannon (*H*)						
LSD						
(I) Season	(J) Season	Mean Difference (I-J)	Std. Error	Sig.	95% Confidence Interval	
					Lower Bound	Upper Bound
aut	win	.6018*	.08444	.000	.4178	.7858
	spr	.0527	.08444	.544	-.1313	.2367
	sum	-.0680	.08444	.436	-.2520	.1160
win	aut	-.6018*	.08444	.000	-.7858	-.4178
	spr	-.5492*	.08444	.000	-.7332	-.3652
	sum	-.6698*	.08444	.000	-.8538	-.4858
spr	aut	-.0527	.08444	.544	-.2367	.1313
	win	.5492*	.08444	.000	.3652	.7332
	sum	-.1207	.08444	.179	-.3047	.0633
sum	aut	.0680	.08444	.436	-.1160	.2520
	win	.6698*	.08444	.000	.4858	.8538
	spr	.1207	.08444	.179	-.0633	.3047
Based on observed means. The error term is Mean Square(Error) = .021.						
*. The mean difference is significant at the .05 level.						

Note:

Season abbreviations: aut = autumn, spr = spring.

Indexes: Shannon (*H*) = Shannon–Wiener's diversity index (*H*);

Pielou_e (*J*) = Pielou's evenness index (*J*);

Margalef (*D*) = Margalef's species richness index (*D*).

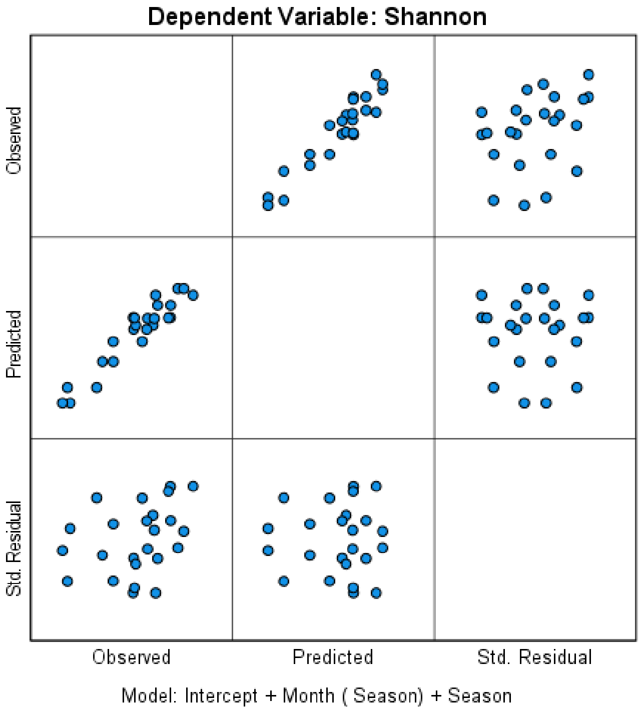

Figure S43. Standardized residual plot of observed vs. predicted Shannon index (*H*).

Table S12. Nested ANOVA of Pielou_e index (*J*).

Tests of Between-Subjects Effects					
Dependent Variable: Pielou_e (*J*)					
Source	Type III Sum of Squares	df	Mean Square	F	Sig.
Corrected Model	.082[a]	11	.007	6.997	.001
Intercept	12.073	1	12.073	11296.571	.000
Month(Season)	.025	8	.003	2.912	.047
Season	.057	3	.019	17.891	.000
Error	.013	12	.001		
Total	12.168	24			
Corrected Total	.095	23			
[a]. R Squared = .865 (Adjusted R Squared = .741)					

Note:

Indexes: Shannon (*H*) = Shannon–Wiener's diversity index (*H*);

Pielou_e (*J*) = Pielou's evenness index (*J*);

Margalef (*D*) = Margalef's species richness index (*D*).

Table S13. Multiple comparisons for Pielou_e index (*J*).

Multiple Comparisons						
Dependent Variable: Pielou_e (J)						
LSD						
		Mean Difference			95% Confidence Interval	
(I) Season	(J) Season	(I-J)	Std. Error	Sig.	Lower Bound	Upper Bound
aut	win	.0601*	.01887	.008	.0190	.1012
	spr	-.0731*	.01887	.002	-.1142	-.0320
	sum	-.0341	.01887	.096	-.0752	.0071
win	aut	-.0601*	.01887	.008	-.1012	-.0190
	spr	-.1332*	.01887	.000	-.1743	-.0921
	sum	-.0942*	.01887	.000	-.1353	-.0530
spr	aut	.0731*	.01887	.002	.0320	.1142
	win	.1332*	.01887	.000	.0921	.1743
	sum	.0390	.01887	.061	-.0021	.0801
sum	aut	.0341	.01887	.096	-.0071	.0752
	win	.0942*	.01887	.000	.0530	.1353
	spr	-.0390	.01887	.061	-.0801	.0021
Based on observed means. The error term is Mean Square(Error) = .001.						
*. The mean difference is significant at the .05 level.						

Note:

Season abbreviations: aut = autumn, spr = spring.

Indexes: Shannon (*H*) = Shannon–Wiener's diversity index (*H*);

Pielou_e (*J*) = Pielou's evenness index (*J*);

Margalef (*D*) = Margalef's species richness index (*D*).

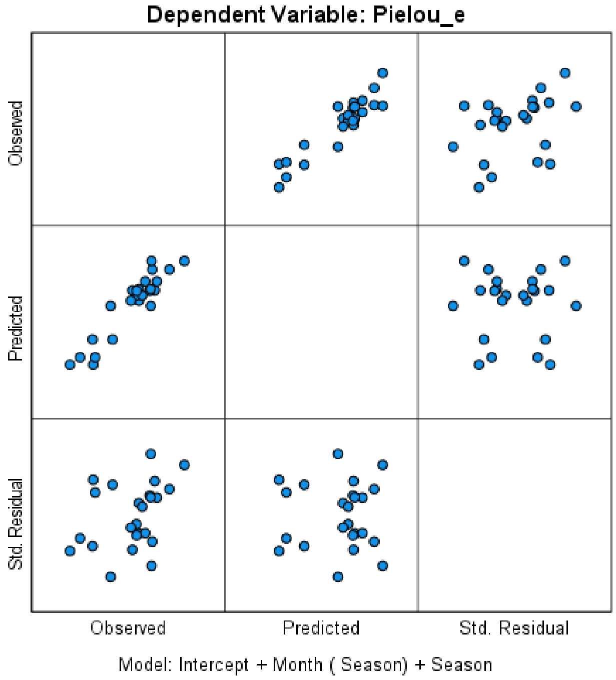

Figure S44. Standardized residual plot of observed vs. predicted Pielou_e index (*J*).

Table S14. Nested ANOVA of Margalef index (*D*).

Tests of Between-Subjects Effects					
Dependent Variable: Margalef (*D*)					
Source	Type III Sum of Squares	df	Mean Square	F	Sig.
Corrected Model	13.484[a]	11	1.226	5.823	.003
Intercept	264.815	1	264.815	1258.037	.000
Month(Season)	7.462	8	.933	4.431	.011
Season	6.022	3	2.007	9.536	.002
Error	2.526	12	.210		
Total	280.825	24			
Corrected Total	16.010	23			
[a]. R Squared = .842 (Adjusted R Squared = .698)					

Note:

Indexes: Shannon (*H*) = Shannon–Wiener's diversity index (*H*);

Pielou_e (*J*) = Pielou's evenness index (*J*);

Margalef (*D*) = Margalef's species richness index (*D*).

Table S15. Multiple comparisons for Margalef index (*D*).

Multiple Comparisons						
Dependent Variable: Margalef (*D*)						
LSD						
(I) Season	(J) Season	Mean Difference (I-J)	Std. Error	Sig.	95% Confidence Interval	
					Lower Bound	Upper Bound
aut	win	.7331*	.26489	.017	.1559	1.3102
	spr	.5661	.26489	.054	-.0110	1.1432
	sum	-.5388	.26489	.065	-1.1159	.0383
win	aut	-.7331*	.26489	.017	-1.3102	-.1559
	spr	-.1670	.26489	.540	-.7441	.4102
	sum	-1.2719*	.26489	.000	-1.8490	-.6947
spr	aut	-.5661	.26489	.054	-1.1432	.0110
	win	.1670	.26489	.540	-.4102	.7441
	sum	-1.1049*	.26489	.001	-1.6820	-.5278
sum	aut	.5388	.26489	.065	-.0383	1.1159
	win	1.2719*	.26489	.000	.6947	1.8490
	spr	1.1049*	.26489	.001	.5278	1.6820
Based on observed means. The error term is Mean Square(Error) = .210.						
*. The mean difference is significant at the .05 level.						

Note:

Season abbreviations: aut = autumn, spr = spring.

Indexes: Shannon (*H*) = Shannon–Wiener's diversity index (*H*);

Pielou_e (*J*) = Pielou's evenness index (*J*);

Margalef (*D*) = Margalef's species richness index (*D*).

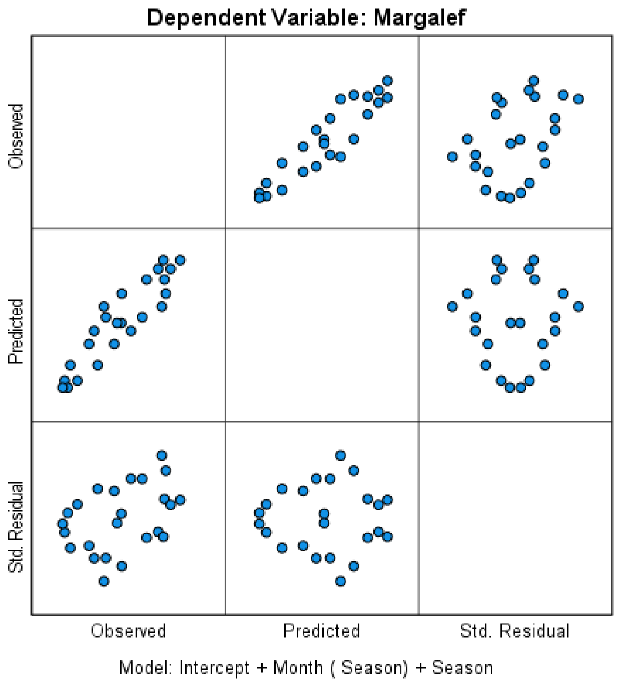

Figure S45. Standardized residual plot of observed vs. predicted Margalef index (*D*)**.**
